# Phosphorylation shapes antigen presentation and immune recognition: mechanistic insights and translational perspectives

**DOI:** 10.3389/fimmu.2026.1824717

**Published:** 2026-08-12

**Authors:** Ramesh Rijal, Alexandre F. Marques

**Affiliations:** School of Biological, Environmental, and Earth Sciences, University of Southern Mississippi, Hattiesburg, MS, United States

**Keywords:** antigen processing, host–pathogen interactions, immune evasion, MHC class I presentation, phosphopeptides, T cell recognition

## Abstract

Phosphorylation is a reversible post-translational modification that dynamically regulates immune responses by reshaping the antigen profiles. Phosphoproteomics and immunopeptidomics studies reveal that phosphorylated peptides (phosphopeptides) are naturally processed and presented by MHC class I and II molecules and can elicit robust T-cell responses. These epitopes influence proteasome cleavage, TAP transport, and MHC loading, defining the immunopeptidome under physiological and pathological states. Structurally, phosphate groups enhance MHC binding and immunogenicity, positioning phosphorylated neoantigens as promising targets for cancer immunotherapy and vaccine development. Beyond adaptive immunity, phosphorylation regulates innate signaling through Toll-like receptors (TLRs) and antigen-presenting cell activation, linking post-translational modifications to immune plasticity. Infections by parasites and bacteria such as *Mycobacterium tuberculosis* exploit phosphoregulation to evade antigen presentation, revealing conserved mechanisms of immune modulation. Integrating phosphoproteomic data with immune profiling may uncover new biomarkers and therapeutic strategies. This review summarizes how phosphorylation shapes antigen presentation and immune recognition across cancer, infection, and inflammation, and outlines future directions for phosphoantigen-based immunotherapy.

## Introduction

Post-translational modifications (PTM) dynamically regulate protein function, localization, and interactions. Among them, phosphorylation is one of the most prevalent and tightly controlled, governing signal transduction, cell-cycle progression, and immune responses. In immunology, phosphorylation has evolved from a basic signaling regulator to a determinant of antigen processing and presentation, now recognized as a potential target for immunotherapy and vaccine design (1–4). Phosphopeptides, peptides bearing phosphorylated serine, threonine, or tyrosine residues, are progressively recognized as immunogenic epitopes presented by MHC class I molecules (5–7). These phosphorylated epitopes often arise from dysregulated kinases or aberrant signaling proteins during oncogenic transformation or infections. As they are less likely than other epitopes to be present in normal cells, they may bypass central tolerance mechanisms. T cells targeting these phosphorylated peptides generally exhibit high avidity and specificity, making them ideal candidates for cancer immunotherapies (7–9). Phosphorylation can enhance peptide–MHC stability and prolong antigen display, thereby increasing the likelihood of productive T cell recognition (5, 10). For the purposes of this review, we use phosphorylation in the antigenic sense primarily to refer to post-translational phosphate addition to serine, threonine, or tyrosine residues within proteins and peptides that may subsequently influence antigen processing, MHC binding, and T-cell recognition, as demonstrated for naturally processed MHC class I-associated phosphopeptides and structurally characterized phosphopeptide–MHC complexes (5, 6, 11). We distinguish these phosphopeptide antigens from phosphate-containing immunomodulators such as monophosphoryl lipid A (MPLA), which is a component of the AS04 adjuvant system and functions as a TLR4-directed innate immune agonist rather than as a phosphopeptide antigen (12, 13). Throughout the manuscript, we therefore separate phosphorylation-specific effects on antigen identity from broader phosphorylation-dependent signaling events that regulate antigen presentation.

**Figure 1:**
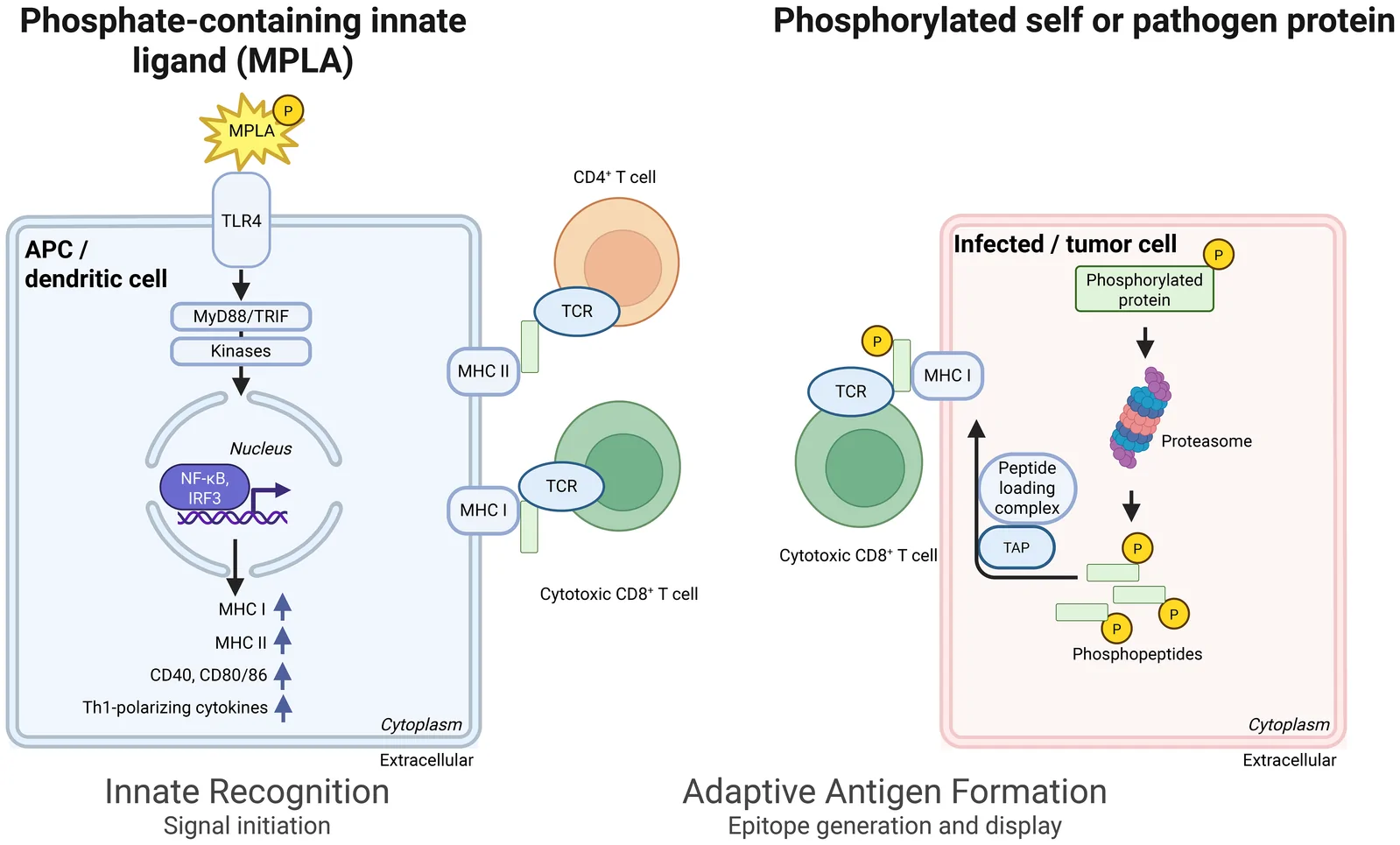
Distinct roles of phosphate-containing innate ligands and phosphorylated peptide antigens in immune recognition. MPLA is shown as a phosphate-containing TLR4 agonist/adjuvant that promotes APC maturation through innate signaling; it is included as an innate immunomodulatory example, not as a phosphopeptide antigen or as the source of phosphate groups in MHC-bound phosphopeptides. In contrast, intracellularly phosphorylated host or pathogen proteins can generate phosphopeptides that enter antigen-processing pathways, bind MHC molecules, and are recognized by TCRs. Depending on phosphosite position and peptide geometry, the phosphate group may contribute to peptide–MHC stabilization and/or remain partially exposed for TCR recognition. Pathogens can also exploit phosphorylation-dependent signaling nodes, including IL-10–STAT3 signaling, phagosome maturation arrest, and CIITA repression, to suppress antigen presentation. Together, these pathways illustrate two related but distinct concepts: phosphate-containing innate immune modulation and phosphorylation-specific antigen presentation.

In addition to adaptive immunity, phosphorylation also plays a role in regulating innate immunity, particularly by promoting activation of pattern recognition receptors (PRRs) (14, 15). An important example is monophosphoryl lipid A (MPLA), a detoxified, phosphate containing derivative of lipopolysaccharide (LPS) derived from Gram-negative bacteria. MPLA, as a TLR4 agonist, initiates TRIF-biased signaling, leading to DC maturation and a Th1-biased immune response (12, 16–18). It has been widely used as an adjuvant in licensed vaccines, including the Cervarix HPV vaccine, demonstrating that phosphate-containing innate agonists can be safe and potent immunomodulators (19, 20). Together, phosphopeptide antigens and phosphate-containing innate agonists illustrate two related but distinct ways in which phosphate chemistry shapes immunity: phosphopeptides can alter antigen identity and T-cell recognition, whereas MPLA functions as a TLR4-directed adjuvant that enhances innate immune activation and downstream adaptive responses (12, 13). This intersection provides a conceptual basis for exploiting phosphate-containing adjuvants and phosphopeptide antigens in vaccine and immunotherapy strategies. As summarized in **Figure 1**, phosphorylation integrates key aspects of innate sensing, antigen processing, and T cell recognition into a unified phospho-immunological axis.

This review highlights how phosphorylation contributes to immune plasticity by modulating antigen presentation, T-cell recognition, and innate signaling, and discusses its translational implications for personalized vaccines and immunotherapies.

## Phosphorylation and antigen processing

Protein phosphorylation profoundly influences antigen processing and presentation by modulating peptide generation, transport, and MHC loading (21, 22). Dysregulation of phosphorylation is a hallmark of both cancer and infection, generating *de novo* phosphorylation-dependent neoantigens (phospho-neoantigens) that are preferentially processed and presented by MHC class I molecules, providing new targets for T-cell recognition (5). It is known that phosphorylation can influence proteasomal cleavage efficiency and substrate processing (23, 24). Depending on the residue’s position and surrounding context, phosphorylation can either accelerate proteasomal degradation or stabilize cleavage sites, shaping which fragments enter the MHC class I pathway. Such cleavage motifs can yield peptides containing phosphorylated residues, which are suitable for MHC class I recognition (25–27). These phosphopeptides are transported by TAP (transporter associated with antigen processing), while phosphorylation may regulate their translocation efficiency. Andersen et al. showed that phosphopeptides derived from oncoproteins are selectively transported into the ER and exhibit distinct binding affinities to MHC-I, with some improved and some diminished, resulting in a repertoire of phospho-specific epitopes (28). Inside the ER, phosphopeptides engage with TAP and associate with tapasin, calreticulin, and other chaperones to form the peptide-loading complex (PLC) (29). Post-translational modifications of peptide-loading components, including TAP phosphorylation, can regulate antigen transport efficiency. For example, X-ray crystallographic analysis of phosphopeptide–HLA-A2 complexes revealed a phosphate anchor that significantly enhances peptide-binding stability and T cell recognition (5, 11). It has also been reported that phosphorylation of PLC components, such as TAP, by p21 (hRSV), regulates the kinetics of antigen presentation (22). Collectively, phosphorylation contributes to antigen diversity, generating an additional layer of immunological plasticity by altering the set of phosphorylated peptides, known as the phosphopeptidome, displayed on MHC molecules, allowing the immune system to identify changes in tumor or infected cells through different phospho-epitopes (11, 30). These changes can impact proteasomal processing, ER translocation, and MHC loading, leading to the presentation of a distinct phosphopeptidome. This plasticity not only broadens the recognition of antigenic targets by tumor-specific T cells but also enables immune surveillance of subtle perturbations in cellular phospho-signaling networks that affect the immune system, including cancer cells (31, 32).

## MHC class I and II presentation of phosphopeptides

Phosphorylated peptides possess distinct biochemical and structural characteristics that critically influence their MHC presentation, immunodominance, and immunogenic potential. This section focuses on the structural and functional principles of phosphopeptide presentation, particularly those elucidated in cancer immunology, where most data are currently available (33, 34). Structural studies indicate that the contribution of phosphorylation to phosphopeptide presentation is context dependent. In some peptide–MHC complexes, the phosphate group contributes to peptide stabilization through interactions within the MHC binding groove, including contacts with positively charged residues, thereby enhancing complex stability (5, 11). In other complexes, the phosphate group remains partially solvent exposed and can participate directly in TCR engagement, thereby altering the antigenic surface recognized by phosphopeptide-specific T cells (35, 36). These roles are therefore not mutually exclusive but vary with phosphosite position, peptide conformation, and HLA allele context (36). Structural studies demonstrate that phosphate groups can function as auxiliary anchor residues, markedly increasing peptide–MHC affinity and stability (5, 34, 36). These epitopes are often derived from deregulated kinase activity in cancer cells, enabling the immune system to recognize “altered-self” signals (9). Immunopeptidomics studies show that phosphopeptides are presented across diverse HLA alleles (A*02:01, B*07:02, B*40:01), with advantages in stability over non-phosphorylated peptides. As a result, they can drive potent CD8^+^ T cell responses, especially when native central tolerance is bypassed (10, 37). Emerging evidence indicates that phosphopeptides are promising neoantigen targets in oncology. In mouse models, immunization with a TRAP1-derived phosphopeptide delayed tumor growth and improved survival (38, 39). Clinical trials in melanoma patients have explored HLA-A2-restricted phosphopeptide vaccines (e.g., from BCAR3 and IRS2), demonstrating safety and immunogenicity. Together, preclinical and clinical data validate phosphorylated epitopes as promising candidates for both personalized and shared tumor-antigen vaccine strategies (40, 41). Although most characterized phosphopeptides are presented via MHC class I, recent work has identified MHC class II-restricted phospho-epitopes that expand the landscape of phosphorylation-dependent immune recognition (8).

## T cell recognition of phosphorylated epitopes

Phosphorylated epitopes are recognized by T cell receptors (TCRs) with exceptional precision, yet their unique chemical features can also enable cross-reactive recognition across different peptide-MHC complexes. This duality makes phospho-epitopes potent activators of adaptive immunity while simultaneously serving as fine modulators of T-cell specificity (42, 43). Phosphorylated epitopes introduce unique chemical features that directly shape TCR engagement. Structural analyses show that exposed phosphate groups extend toward TCR complementarity-determining regions, enabling electrostatic and hydrogen-bond interactions that confer high specificity (5, 35). Even single phosphorylation events can generate sufficient structural contrast to elicit strong CD8^+^ T cell responses while bypassing central tolerance (44–46). Despite this specificity, certain phosphopeptides can be recognized by multiple TCRs, suggesting structural conservation among TCR contact regions (47). For example, the phosphopeptides TAXp5-GAG phosphorylated at Serine-5 are recognized by multiple TCR clonotypes, including “public” or shared TCRs detected across different individuals (48). Such cross-reactivity expands immune coverage but introduces a delicate balance between broad surveillance and the potential for autoreactivity. Phosphorylation can both reveal novel neoepitopes and obscure existing ones. In cancers, mutations increasing kinase activity may generate new phospho-neoantigens not present in healthy tissues, which become targets of CD8^+^ T cells (49, 50). Conversely, tumor-mediated phosphorylation within proteasome cleavage sites can prevent peptide generation, thereby allowing immune evasion (51, 52). Additionally, some pathogens further exploit phosphorylation mimicry to evade immune detection or induce tolerance, illustrating an evolutionary convergence between microbial immune evasion and host signaling networks (53–55). Several immunological properties discussed here, including MHC restriction, polyclonal TCR recognition, clonal selection, and cross-reactive recognition, are shared with many conventional peptide antigens and are not unique to phosphopeptides. What is more specific to phosphopeptide antigens is the altered chemistry introduced by the phosphate group itself, which can modify peptide–MHC stability, reshape exposed TCR-contact surfaces, and reveal disease-associated signaling states that are not captured by amino acid sequence variation alone (5, 11, 35, 36). Together, these findings highlight phosphorylation as a double-edged sword, enhancing antigen visibility in some contexts while enabling immune evasion in others, a duality also reflected in innate regulation.

Tolerance to phosphopeptides is unlikely to be absolute. Physiologically presented phosphopeptides may contribute to central and peripheral tolerance when they are stably generated and displayed during immune education, consistent with general principles of T-cell tolerance to self-antigens (46). However, many disease-associated phosphopeptides arise under altered kinase activity, inflammatory stress, oncogenic transformation, infection, or tissue-restricted signaling contexts that may change their abundance, timing, phosphorylation stoichiometry, or HLA presentation (10, 40, 56). Such epitopes may therefore behave as altered-self antigens: not fully foreign, but not necessarily represented at sufficient abundance or in the appropriate context during tolerance induction. This framework helps explain how phosphopeptides can be immunogenic in cancer while also remaining relevant to autoimmune risk.

## Phosphorylation-dependent signaling that modulates antigen presentation

Phosphorylation is a central regulator of innate immune signaling, particularly in pathways triggered by toll-like receptor (TLR) engagement, where it shapes antigen presentation and dendritic cell or macrophage function (57). Upon recognition of pathogen-associated molecular patterns (PAMPs), TLRs, especially TLR4, recruit adaptor proteins such as MyD88, TIRAP, TRIF, and TRAM, which, in turn, activate kinases such as IRAK1/4 and TBK1 (58–60). These kinases phosphorylate key downstream effectors, including NF-κB, mitogen-activated protein kinases (MAPKs), and IRF3, ultimately inducing pro-inflammatory cytokine production and IFN responses. Phosphoproteomics analysis has mapped thousands of stimulus-specific phosphorylation events downstream of TLR activation, revealing that innate signaling is both transient and highly modular (61–63). Beyond cytokine induction, phosphorylation modulates antigen-processing machinery and co-stimulatory molecule expression in APCs: a) Kinase-regulated maturation: Dendritic cells stimulated via TLR4 (e.g., by monophosphoryl lipid A) display coordinated phosphorylation of proteins involved in antigen-processing and trafficking, enhancing upregulation of MHC and co-stimulatory molecules such as CD40, CD80, and CD86 (64, 65); b) Phosphoproteome shifts in DCs and macrophages: Phosphoproteomic profiles in these cells demonstrate phosphorylation-dependent activation of pathways (e.g., JAK–STAT, IL-12 regulation), refining antigen presentation and cytokine output (66, 67); c) Adapter phosphorylation and antigen load: Scaffold proteins such as phosphorylated trafficking adaptor proteins (pTRAPs) undergo phosphorylation to recruit downstream effectors, controlling cross-presentation and endosomal antigen routing (68, 69). Collectively, these outcomes establish phosphorylation as a dynamic bridge between pathogen detection and antigen presentation (**Figure 1**). By tuning APC maturation and peptide display, phosphorylation determines the magnitude and quality of T-cell activation. Deciphering these phospho-regulatory networks offers new strategies to amplify vaccine efficacy or restrain hyper-inflammatory states.

## Clinical implications and translational perspectives of phosphopeptides

The translational relevance of phosphorylated peptides has rapidly expanded, encompassing cancer immunotherapy, autoimmunity, infectious diseases, and diagnostics (11, 38, 70–72). Emerging clinical data demonstrate the feasibility and immunogenic potential of tumor-associated phosphopeptides as vaccine candidates (73, 74). These vaccines, particularly when formulated with adjuvants such as poly-ICLC or Freund’s, are advancing into trials for hematological malignancies and colorectal cancer (9). Preclinical models reinforce these findings, showing enhanced anti-tumor immunity and survival. Phosphopeptides therefore present a promising strategy for both shared and personalized neoantigen vaccine platforms, particularly in tumors with low mutational burden (75, 76). Phosphopeptides also hold great promise as diagnostic and therapeutic tools. Biomarkers: Immunopeptidomic surveys reveal that a measurable fraction of MHC-bound peptides carry post-translational modifications (PTMs), including phosphorylation (77, 78). These phosphopeptides can serve as biomarkers for dysregulated signaling in tumors or immune disorders. Adoptive Cell Therapy: T cells specific to phospho-neoantigens are being explored for precision immunotherapies, particularly where conventional mutation-derived neoantigens are scarce (3, 79). Next-Generation Vaccines: Multitope platforms incorporating both mutated and PTM-modified antigens are being developed to broaden immune coverage and enhance efficacy (80). These applications are made possible by recent advances in high-resolution mass spectrometry, which enable sensitive and specific detection of low-abundance phosphopeptides across various tissue types (81, 82).

**Figure 2:**
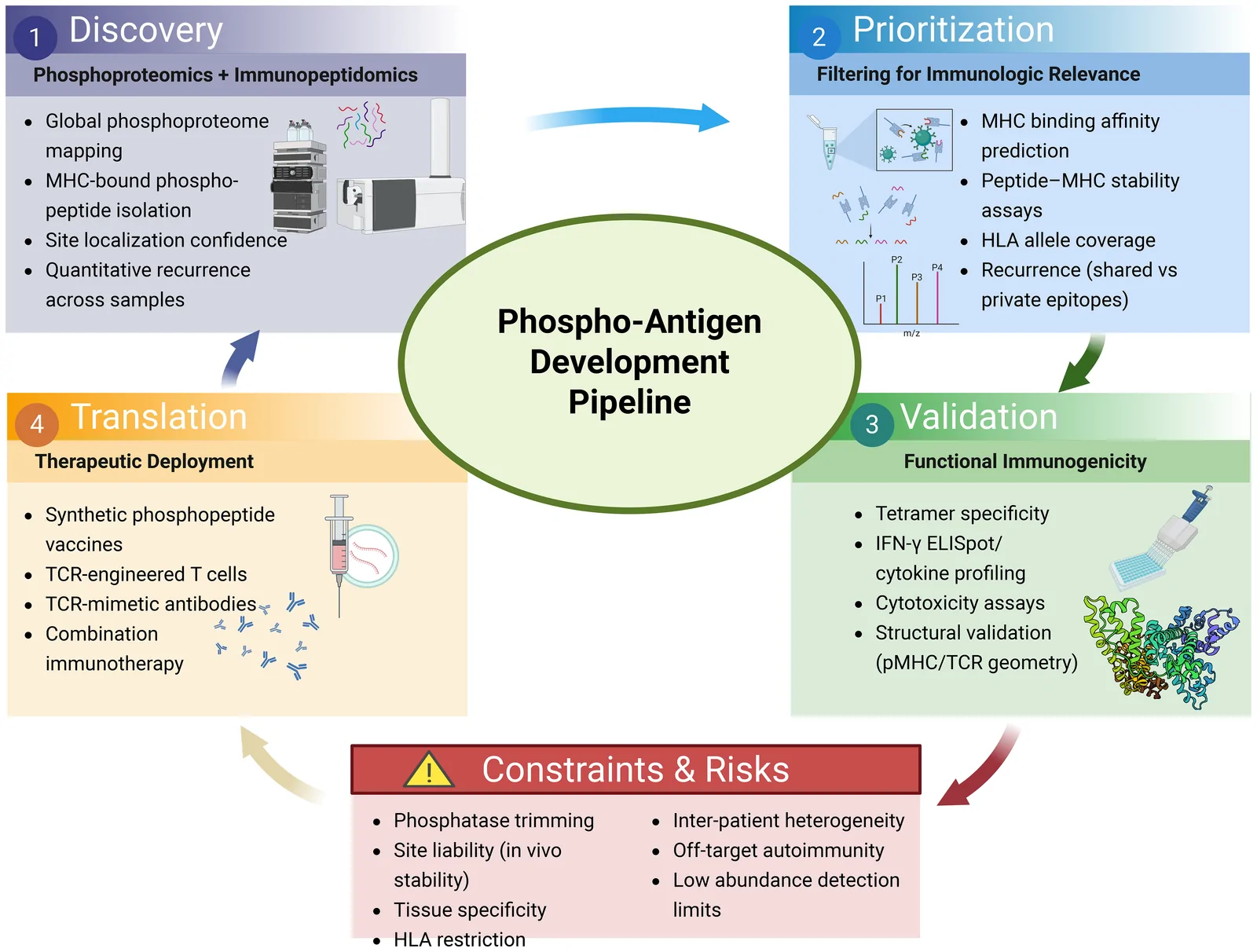
Roadmap from phosphoproteome discovery to phosphopeptide-directed immunotherapy. Discovery integrates phosphoproteomics and immunopeptidomics to identify phosphorylated peptides presented on MHC molecules. Prioritization ranks candidates by HLA match, disease specificity, recurrence across patients, pathway linkage, predicted peptide–MHC stability, and feasibility as shared or personalized targets. Validation includes tetramer binding, ELISpot or cytokine assays, cytotoxicity assays, structural assessment of pMHC–TCR interactions, and cross-reactivity or normal-tissue screening. Translational modalities include synthetic phosphopeptide vaccines, TCR-engineered T cells, and TCR-mimetic antibodies. RNA- or minigene-based approaches should be considered conditional, because they encode precursor sequences and rely on endogenous cellular phosphorylation; therefore, phosphopeptide generation and presentation must be experimentally validated. Key constraints include phosphosite lability, low abundance, HLA restriction, inter-patient heterogeneity, tissue specificity, and off-target autoreactivity.

In oncology, phosphopeptide profiling may become clinically informative when it identifies recurrent, HLA-matched phosphopeptide targets linked to dysregulated signaling pathways, particularly in tumors with limited canonical neoantigen burden (6, 10, 40). In that setting, immunopeptidomic detection could help prioritize phosphopeptide vaccine candidates, TCR-engineered T-cell targets, or TCR-mimetic antibody strategies, and may also help monitor pathway rewiring or immune escape during treatment (40, 41). However, these applications remain investigational and should not be interpreted as support for phosphopeptide burden alone as a current clinical decision metric. Similarly, in autoimmune diseases, phosphopeptide signatures could aid in early diagnosis and stratification of patients based on disease severity or progression risk (83, 84). From a therapeutic perspective, the development of TCR-engineered T cells targeting phospho-epitopes opens new avenues for adoptive cell transfer strategies. These phospho-specific T cells have shown high specificity and cytotoxic potential against cancer cells while sparing healthy tissues, due to the restricted expression of phosphorylated antigens (85, 86). Moreover, platforms combining phosphopeptide vaccines with immune checkpoint inhibitors may synergize to overcome tumor-induced immunosuppression and enhance effector T cell function (87). Collectively, these advances establish a translational pipeline from phosphoproteomic discovery to phospho-specific immunotherapy (**Figure 2**).

## Quantitative and functional relevance of phosphopeptides in the immunopeptidome

Although phosphorylated peptides constitute only a relatively small fraction of the total MHC-associated immunopeptidome, accumulating evidence suggests that they may possess disproportionately high immunological relevance in specific pathological contexts, particularly in cancer and chronic infection (56, 88, 89). Immunopeptidomic studies have identified phosphorylated peptides across multiple HLA alleles, with some reports estimating that post-translationally modified peptides represent approximately 0.5–5% of the detectable MHC-bound peptide repertoire, depending on the biological system, enrichment strategy, and analytical sensitivity employed (78, 90). Despite their relatively low abundance, phosphopeptides can exhibit enhanced peptide–MHC stability through phosphate-mediated auxiliary anchor interactions and may generate highly specific T-cell responses capable of recognizing altered signaling states in diseased cells. Structural and functional studies further demonstrate that phosphorylation can substantially alter TCR contact chemistry, peptide affinity, and immunogenicity compared to non-phosphorylated counterparts. Nevertheless, important limitations remain (9, 91). The prevalence and reproducibility of phosphopeptide presentation across patients, tissues, and disease states are still incompletely characterized, and standardized quantitative comparisons of immunogenic effect sizes remain limited across studies. These challenges highlight the need for larger-scale immunopeptidomic datasets and standardized analytical frameworks to better define the biological and translational significance of phosphopeptide antigens (92, 93).

Consistent with these estimates, additional immunopeptidomic surveys place the presented phosphopeptide fraction within a comparably low range that varies with cell type, stimulation state, and mass spectrometry depth (119–121). Despite their low abundance, several phosphopeptides have demonstrated enhanced immunogenicity compared with their non-phosphorylated counterparts, including increased MHC class I binding stability and stronger CD8+ T-cell activation in both cancer and infectious disease contexts (122). In some experimental systems, phosphorylation increased peptide–MHC complex stability by several fold and substantially amplified IFN-γ-producing T-cell responses. However, quantitative clinical data remain limited, particularly regarding prevalence across patient cohorts, reproducibility between studies, and objective response metrics *in vivo* (123, 124).

## Technical limitations in phosphopeptide identification

Despite major advances in phosphoproteomics and immunopeptidomics, significant technical limitations continue to complicate the identification and interpretation of phosphorylated epitopes (94, 95). Phosphosites are inherently labile and susceptible to phosphatase-mediated dephosphorylation during tissue collection, sample preparation, and peptide processing, potentially leading to underrepresentation of phosphopeptides in downstream analyses. In addition, the relatively low abundance and transient nature of many phosphopeptides challenge the sensitivity and dynamic range of current mass spectrometry platforms, particularly for MHC-bound immunopeptidome studies (96–98). Accurate phosphosite localization also remains difficult, as incorrect assignment of phosphorylation sites may generate false-positive identifications or misinterpretation of immunogenic epitopes. Furthermore, commonly used enrichment strategies, including TiO_2_ and immobilized metal affinity chromatography (IMAC), introduce selective biases toward specific phosphopeptide populations and may incompletely capture the true phosphoproteome landscape (99–101). Immunopeptidomic analyses are additionally influenced by HLA allele-dependent presentation biases, since peptide-binding motifs and phosphopeptide compatibility vary substantially among MHC alleles. Together, these technical and biological constraints highlight the need for improved phosphopeptide stabilization methods, higher-sensitivity analytical platforms, and standardized computational pipelines to more accurately define the *in vivo* phospho-immunopeptidome (102–104).

## Integration of phosphoproteomics and immunopeptidomics

Recent advances in immunopeptidomics have substantially expanded our understanding of how phosphorylated peptides are naturally processed and presented by MHC molecules across diverse biological contexts (104–106). In contrast to conventional phosphoproteomics, which primarily catalogs intracellular phosphorylation events, immunopeptidomics directly interrogates the subset of phosphopeptides that successfully enter antigen-processing pathways and are displayed on the cell surface for immune surveillance (37). Multiple studies have now identified MHC-bound phosphopeptides across several HLA alleles, including HLA-A*02:01, HLA-B*07:02, and HLA-B*40:01, demonstrating that phosphorylation-dependent antigen presentation is not restricted to a single MHC background (107, 108). Importantly, several phosphopeptides have been reproducibly detected across independent tumor samples and among different patients, suggesting the existence of partially shared phospho-neoantigen landscapes. Comparative immunopeptidomic analyses further indicate that phospho-neoantigens may complement canonical mutation-derived neoantigens by revealing dysregulated signaling states that are not captured through genomic sequencing alone (38, 109, 110). In some tumors with relatively low mutational burden, phosphorylation-derived epitopes may therefore provide an additional layer of immunological targetability. Nevertheless, substantial variability remains across datasets due to differences in HLA composition, tissue origin, phosphopeptide enrichment strategies, and analytical sensitivity. Continued integration of phosphoproteomics with high-resolution immunopeptidomics will be essential to define the reproducibility, abundance, and translational relevance of phosphopeptide presentation *in vivo* (10, 111, 112).

## Phosphopeptide presentation across diverse biological contexts

Although cancer immunology currently represents the most extensively studied context for phosphopeptide antigen presentation, emerging evidence suggests that phosphorylation-dependent immune recognition also plays important roles in viral infection, autoimmunity, and physiological tissue homeostasis (9). Several viruses, including Epstein–Barr virus, cytomegalovirus, hepatitis viruses, and SARS-CoV-2, are known to extensively remodel host phosphorylation networks during infection, potentially generating altered phosphopeptide repertoires that may contribute to antiviral immune surveillance (113–115). Likewise, aberrant phosphorylation has been implicated in autoimmune diseases such as rheumatoid arthritis, systemic lupus erythematosus, and multiple sclerosis, where dysregulated kinase signaling and post-translationally modified self-antigens may contribute to the breakdown of immune tolerance (116, 117). Importantly, phosphorylated peptides are not exclusively disease-associated and can also be physiologically presented in normal tissues as part of routine cellular turnover, signaling regulation, and immune surveillance mechanisms. However, the abundance, stability, and immunogenicity of physiological phosphopeptides in healthy tissues remain incompletely characterized (5, 27, 118). In the context of infectious diseases, phosphorylation-mediated immune modulation extends beyond intracellular bacteria and parasites and likely contributes broadly to host–pathogen interactions across viral, fungal, and protozoan systems. Nevertheless, the current literature remains disproportionately concentrated within cancer immunopeptidomics, reflecting both the rapid expansion of tumor neoantigen research and the relative scarcity of experimentally validated phosphopeptide datasets in non-malignant conditions.

## Phosphopeptides in parasitic infections

While phosphopeptide-based strategies have largely focused on cancer, recent studies highlight their emerging relevance in parasitic infections (125, 126). Phosphorylated epitopes derived from intracellular parasites can be processed by the host antigen-presenting machinery, loaded onto MHC class I molecules, and displayed on the surfaces of infected cells. In *Trypanosoma cruzi*, Sec22b-dependent cross-presentation contributes to CD8^+^ T-cell priming, illustrating the importance of intracellular trafficking routes in antigen presentation during parasitic infection (127). However, Sec22b is not itself evidence of a phosphorylation-specific antigenic mechanism, and direct demonstration of pathogen-derived phosphopeptide presentation in this setting remains limited. Similarly, in *Toxoplasma gondii* and *Leishmania* spp., phosphorylation influences proteasomal cleavage preferences and peptide stability, thereby expanding the diversity and immunogenicity of parasite-derived epitopes presented to T cells (128, 129). These phospho-neoantigens, which may be absent from uninfected host cells, represent highly specific targets for vaccine development and immune monitoring in parasitic diseases, bridging key aspects of cell signaling and host-pathogen immunobiology. Emerging preliminary data from experimental models further support this concept, although these findings require independent validation. In our own exploratory analyses (unpublished data), in silico evaluation of Tc85pep, a peptide derived from *Trypanosoma cruzi* trans-sialidase, revealed threonine residues predicted to be phosphorylated at conserved PKA and PKG motifs, along with high binding affinity to murine and human MHC class I and II alleles. Structural modeling suggested potential interactions with the TLR4/MD2 complex. Structural insights into TLR4–MD2 recognition have been previously described (130), raising the possibility of innate immune engagement. In our preliminary studies (unpublished data), a Qβ-Tc85pep conjugate in TLR4-deficient macrophages showed TLR4-dependent nitric oxide production. In immunized mice, Qβ-Tc85pep induced a protective immune response against *T. cruzi*, reducing parasitemia and cardiac pathology, and eliciting a mixed Th1/Th2/Th17 cytokine profile. These preliminary observations suggest that Tc85pep may function as a multifunctional phospho-epitope that engages both innate and adaptive immunity. This highlights phosphorylated parasite antigens as promising candidates for vaccine development in Chagas disease and other parasitic infections. Although intracellular pathogens extensively manipulate host phosphorylation networks to alter phagosome maturation, autophagy, cytokine signaling, and MHC expression, direct evidence linking these pathways to defined pathogen-derived phosphopeptides presented on MHC molecules remains limited. Therefore, the current literature supports a strong role for phosphoregulation in shaping antigen presentation during infection, but the identification of bona fide pathogen-associated phosphopeptide epitopes by immunopeptidomics remains an emerging and underexplored area (131, 132).

## Phosphoregulation-based disruption of antigen presentation by intracellular pathogens

Intracellular pathogens such as *Mycobacterium tuberculosis* (Mtb) and protozoan parasites such as *Leishmania* spp. and *Toxoplasma gondii* have evolved convergent strategies to subvert host antigen presentation by hijacking host phosphorylation-dependent pathways. These mechanisms impair multiple stages of antigen processing and presentation, from phagosome maturation and autophagy to MHC loading and T cell activation, thereby enabling the microbes to evade immune detection. Below, we outline how bacterial and parasitic intracellular pathogens exploit phosphoregulation to disrupt both MHC class II and class I antigen presentation via transcriptional, epigenetic, trafficking, and signaling mechanisms, often using specialized kinases or phosphatases as tools.

## Arresting phagosome maturation

Pathogens often arrest normal phagosome maturation to avoid lysosomal destruction. Mtb achieves this by co-opting host phosphatase and kinase pathways. Upon entry into macrophages, Mtb recruits the host actin-binding protein coronin-1 to the phagosomal membrane, triggering Ca²^+^ flux and activation of the phosphatase calcineurin. The resulting calcineurin signaling blocks phagosome–lysosome fusion, allowing the bacteria to survive inside a stalled vacuole (133). In coronin-1–deficient macrophages, this blockade fails; calcineurin is not activated, and phagosomes proceed to fuse with lysosomes, leading to mycobacterial killing (133). Mtb also secretes a eukaryotic-like Ser/Thr protein kinase, PknG, that prevents phagosomal acidification and fusion by targeting host Rab7/Rab7L1 GTPase trafficking pathways (134). Deletion or pharmacologic inhibition of PknG causes bacteria to be routed promptly to lysosomes for destruction (134). In parallel, Mtb exports secreted phosphatases that subvert phagosome maturation. The secreted lipid phosphatase SapM hydrolyzes phosphatidylinositol-3-phosphate, thereby disrupting phagosome–late endosome fusion and stalling phagolysosome maturation (135). Likewise, the secreted tyrosine phosphatase PtpA binds to the V-ATPase proton pump complex and excludes it from phagosomal membranes, preventing acidification of the compartment (136). Through these concerted actions, Mtb blocks membrane fusion and neutralizes the phagosomal pH, creating a hospitable, non-acidified vacuole in which it can persist.

Protozoan parasites deploy analogous tactics to modulate phagosome maturation via host phosphoregulation. For example, *Leishmania donovani* promastigotes, after being phagocytosed by macrophages, avoid destruction by using their surface lipophosphoglycan (LPG) to dysregulate host kinase signaling. LPG inserts into the phagosomal membrane and prevents normal recruitment and activation of protein kinase C (PKC) on the nascent phagosome (137). As a result, periphagosomal F-actin fails to depolymerize, and an F-actin “cage” persists around the phagosome, physically blocking lysosomal fusion. By inhibiting PKCα-dependent actin remodeling, *Leishmania* effectively delays phagosome maturation, paralleling the calcineurin-mediated fusion block observed in Mtb. Other parasites achieve similar outcomes by actively modifying host signaling molecules. *Toxoplasma gondii*, for instance, resides in a non-fusogenic parasitophorous vacuole and secretes rhoptry kinase ROP18, which binds to and phosphorylates host immunity-related GTPases (IRGs) (138). This modification disables the IRGs that would normally attack the vacuole, thereby preventing the vacuole’s disruption and lysosomal exposure (138). In mice, ROP18-mediated IRG phosphorylation is essential for virulent *T. gondii* strains to avoid vacuolar clearance by IFN-γ-activated macrophages (138). Thus, across kingdoms, intracellular bacteria and parasites converge on the strategy of manipulating host kinases and phosphatases at the phagosome interface to arrest maturation, buying time to establish their intracellular niche.

## IL-10–STAT3–mTOR axis: suppression of autophagy and antigen processing

This section focuses on phosphorylation-dependent signaling only where it alters antigen-processing capacity, autophagy-dependent antigen routing, MHC expression, or T-cell priming, rather than treating receptor phosphorylation as inherently antigen-specific. In addition to blocking phagolysosome fusion, many pathogens interfere with autophagy and related antigen processing pathways through phospho-regulated immunosuppressive circuits. A central player is the anti-inflammatory cytokine interleukin-10 (IL-10), whose production is often driven by pathogen-activated kinase signaling in infected antigen-presenting cells. Virulent Mtb skews infected macrophages toward an IL-10^high^/IL-12^low^ phenotype by engaging pattern recognition receptors and downstream kinases. For example, Mtb cell wall components such as mannosylated lipoarabinomannan (ManLAM) and the 19-kDa lipoprotein signal through TLR2 and MAPK p38, rapidly inducing IL-10 while suppressing IL-12 production in macrophages (139, 140). This early IL-10 upregulation is linked to activation of the PI3K/Akt pathway and concomitant inhibition of GSK-3β, which together favor CREB-dependent IL-10 transcription over pro-inflammatory NF-κB responses. This establishes a feedback loop that suppresses macrophage microbicidal activity, autophagic flux, and antigen processing (139). IL-10 engages its receptor on macrophages, activating JAK1 and TYK2, which phosphorylate STAT3 and initiate an anti-inflammatory program. A key outcome of IL-10–STAT3 signaling is the suppression of autophagy: IL-10R/STAT3, via activation of Akt and mTOR, inhibits starvation-induced autophagy and autolysosome formation (141). Consistent with this, neutralizing IL-10 in Mtb-infected human macrophages allows phagosome maturation and autophagy to proceed, whereas the presence of IL-10 blocks phagosomal maturation and permits bacterial survival (142). Mtb exploits this axis by using its secreted epigenetic modifier Eis (enhanced intracellular survival protein) to drive IL-10 production: Eis acetylates histones at the *IL10* promoter, upregulating IL-10 and thereby activating Akt/mTOR to suppress autophagy and aid bacterial persistence (143).

In antigen-presenting cells, IL-10 further impairs antigen processing by disrupting MHC class II trafficking and surface expression. IL-10 signaling causes newly synthesized MHC-II–peptide complexes to be sequestered into intracellular vesicles rather than displayed on the plasma membrane. Additionally, IL-10 induces the E3 ubiquitin ligase MARCH1 in monocytes (144, 145), which ubiquitinates MHC-II and mediates its internalization and degradation. IL-10–activated STAT3 also upregulates suppressors such as SOCS3 and the NF-κB inhibitor BCL-3, and it engages the inositol phosphatase SHIP1; together, these pathways curb NF-κB–driven pro-inflammatory outputs and reduce IL-12 and TNF-α production. Through these mechanisms, an IL-10–rich environment potently blunts the macrophage’s ability to process antigens and present them to T cells.

Parasitic infections likewise harness host phospho-signaling to induce an IL-10–dominated state that dampens autophagy and antigen processing. In *Leishmania* spp., both surface and secreted parasite molecules trigger signaling that elevates IL-10 while suppressing pro-inflammatory pathways. For instance, *Leishmania* LPG not only perturbs phagosome maturation, as noted above, but also suppresses IL-1β and other inflammatory genes in monocytes. *Leishmania* also secretes GP63, a metalloprotease, which activates host protein tyrosine phosphatases (including SHP-1) that silence macrophage MAPK and JAK/STAT signaling (146). During *Leishmania* infection, macrophage CD40 signaling is skewed toward extracellular signal-regulated kinase (ERK) (promoting IL-10) over p38 (which would promote IL-12); experimentally forcing the balance back toward p38 (or inhibiting ERK) restores IL-12 production and improves parasite control. A key virulence axis is the activation of host SHP-1 by *Leishmania*: GP63 cleaves and activates SHP-1, and active SHP-1 directly dephosphorylates the TLR/IL-1R adaptor IRAK-1 (147), thereby throttling NF-κB signaling and IL-12 induction. Other protozoa use comparable tactics. In *T. gondii*, the rhoptry kinase ROP16 phosphorylates and activates host STAT3, driving an IL-10^high/IL-12^low phenotype in infected dendritic cells and macrophages. This skews the myeloid cells toward an anti-inflammatory, non-autophagic state that favors parasite persistence. Consistent with the importance of IL-10 in these infections, blocking IL-10 signaling or genetically ablating IL-10 markedly enhances pathogen clearance *in vivo*: IL-10–deficient mice infected with Mtb display earlier, stronger Th1 responses and reduced bacterial loads (139), and *Leishmania*-infected mice treated with anti-IL-10R antibodies achieve more effective parasite control. Thus, by subverting host kinase pathways to induce IL-10 and related suppressors, intracellular microbes create a permissive intracellular environment with reduced antigen processing.

## Phosphorylation-coupled metabolic reprogramming in antigen presentation

Antigen-presenting cell activation is metabolically demanding, and phosphorylation-dependent signaling is the switch that couples metabolic state to antigen-processing capacity. Toll-like receptor engagement triggers rapid Akt-mTORC1-driven glycolytic commitment in dendritic cells, which supplies the lipid and biosynthetic precursors required for antigen-processing organelle expansion and MHC trafficking during maturation (148, 149), and this Akt-mTORC1-glycolysis axis is similarly required for the enhanced antigen-presenting potency of highly stimulatory dendritic cell subsets (150). A parallel hypoxia-inducible factor-1 (HIF-1)-dependent glycolytic program governs macrophage polarization toward a proinflammatory, antigen-presentation-competent M1-like state during Mtb infection (151), and this metabolic checkpoint is a direct target of pathogen subversion: Mtb actively decelerates macrophage glycolytic and oxidative metabolism to favor a quiescent bioenergetic state that limits antimicrobial and antigen-presenting function (152), while *Leishmania* spp. similarly reprogram macrophage AMP-activated protein kinase (AMPK)-mTOR-peroxisome proliferator-activated receptor gamma (PPARγ) metabolic signaling to sustain a permissive, immunosuppressive host-cell phenotype (153). These findings indicate that the same phosphorylation-regulated metabolic checkpoints (Akt-mTORC1, AMPK, and HIF-1) that enable effective antigen presentation in activated APCs are recurrently exploited by the intracellular pathogens discussed above to blunt antigen processing and downstream T-cell priming.

## CIITA repression and MHC class II silencing

Ultimately, the above strategies lead to defective antigen presentation on MHC molecules, thereby blunting T-cell responses. Intracellular pathogens can hinder MHC class II antigen presentation at multiple levels by modulating host cell phosphoregulation. In Mtb-infected macrophages, surface MHC-II expression and antigenic peptide loading are often downregulated as a consequence of altered signaling. One well-characterized mechanism involves the 19-kDa mycobacterial lipoprotein (LpqH) engaging TLR2, which not only drives IL-10 production but also induces repressive isoforms of the transcription factor C/EBPβ that shut off the master regulator of MHC-II genes (140). Specifically, TLR2 signaling via the 19-kDa antigen increases expression of C/EBPβ isoforms, particularly the inhibitory LIP form, as well as LAP, which bind to the CIITA gene promoters, preventing CIITA transcription (154). The result is a failure to upregulate MHC-II in infected macrophages even in the presence of IFN-γ. This effect is dependent on downstream phosphorylation events; for example, p38 MAPK activation downstream of TLR2 leads to C/EBPβ phosphorylation and favors the production of the LIP isoform, which correlates with CIITA suppression (154). In addition to this transcriptional block, Mtb delivers secreted effectors that epigenetically silence MHC-II expression. Repression of CIITA, whether by altered transcription factor signaling or by histone modification, leaves infected macrophages with markedly reduced MHC-II antigen-presenting capacity (154).

Compounding this, Mtb’s interference with endosomal maturation further reduces the generation of antigenic peptides for MHC-II. Impaired phagosome acidification, caused by factors such as PtpA and SapM, results in bacterial proteins being poorly degraded into peptides that can be loaded onto MHC-II (136). Consistent with this, dendritic cells exposed to the Mtb 19-kDa lipoprotein show diminished processing of phagosomal antigens for MHC-II presentation (154). The net outcome is a profound decrease in CD4^+^ T-cell activation, allowing the pathogen to persist. Mtb further upregulates host immunoinhibitory checkpoints via phosphosignaling, for example, the IL-10/STAT3 axis and ERK pathway induce the expression of programmed death-ligand 1 (PD-L1) on infected macrophages, which delivers inhibitory signals to T cells. By both reducing antigen presentation and increasing T-cell inhibitory signals, virulent mycobacteria create a multifaceted block to the CD4^+^ T-cell response.

Analogous interference in MHC-II antigen presentation is observed in chronic parasitic infections. In *Leishmania*-infected macrophages, the IL-10/SHP-1 milieu discussed above leads to markedly reduced MHC-II expression and antigen-presenting function. The molecular basis lies in disrupted IFN-γ signaling. Activated SHP-1 dephosphorylates JAK2, STAT1, and other components of the IFN-γ receptor pathway, preventing the transcriptional upregulation of CIITA and MHC-II that normally occurs during macrophage activation. The parasite-induced decrease in NF-κB and nuclear factor of activated T cells (NFAT) activation, via mechanisms including GP63 and LPG, results in lower transcription of *CIITA* and MHC-II genes, mirroring the TLR2–CIITA suppression seen in tuberculosis. The outcome is essentially an “antigen presentation paralysis” in *Leishmania*-harboring cells, in which they fail to upregulate MHC-II and co-stimulatory molecules, thereby yielding poor CD4^+^ T-cell responses and allowing persistent infection. Similarly, *T. gondii* can modulate host transcription factors to downregulate MHC expression. The dense-granule effector *T. gondii* inhibitor of STAT1 (TgIST) is secreted into the host-cell nucleus, where it binds to phosphorylated STAT1 and recruits host chromatin repressors, blocking the transcription of STAT1-dependent genes, including *CIITA* and immunoproteasome subunits (155). This TgIST-mediated functional inactivation of STAT1 in infected cells, akin to a continuous SHP-1 effect or a mimic of STAT1 being “turned off”, prevents IFN-γ from licensing the cell to present antigens on both MHC-II and MHC-I (155). In these ways, disparate pathogens, bacteria and protozoa, both leverage host phosphorylation-controlled pathways to achieve a common goal to diminish the generation and display of antigenic peptides on MHC-II, thus escaping detection by CD4^+^ T cells.

## Suppression of MHC class I cross-priming

Although CD4^+^ T cells are often the primary effectors against these intracellular pathogens, many also take steps to undermine MHC class I antigen presentation and CD8^+^ T-cell responses. Mtb primarily resides within vacuoles, so its antigens have limited access to the cytosolic MHC-I processing machinery. Some bacterial proteins do escape into the cytosol or are released when an infected cell dies, and those antigens can be cross-presented by dendritic cells. However, Mtb actively interferes with these cross-presentation routes. Notably, the same 19-kDa lipoprotein that inhibits MHC-II antigen processing has also been reported to reduce peptide generation via the vacuolar MHC-I pathway (140). Moreover, Mtb infection is known to inhibit apoptosis of its host macrophage by activating pro-survival signaling, in large part through the IL-10/STAT3 axis and PI3K/Akt, which upregulate anti-apoptotic factors (156). By preventing the infected cell’s apoptosis, the bacterium minimizes a key pathway for antigen cross-priming: normally, infected cells undergoing apoptosis release antigen-rich apoptotic bodies that are engulfed by dendritic cells, leading to MHC-I cross-presentation of those antigens. Mtb-infected macrophages kept alive in a pseudo-active state via continuous Akt and STAT3 signaling release far fewer apoptotic vesicles for dendritic cells to capture. Consequently, CD8^+^ T cells are less efficiently primed. In line with this, virulent Mtb strains that strongly activate these survival pathways induce weak CD8^+^ T-cell responses, whereas experimental treatments that promote infected macrophage apoptosis (or that block IL-10/PI3K signals) improve cross-priming of CD8^+^ T cells.

Mtb’s own kinases and phosphatases also likely skew the endosomal and cytosolic trafficking systems in ways that diminish MHC-I antigen presentation. For example, the Mtb kinase PknG and phosphatase PtpB discussed earlier not only block phagosomal maturation but may also alter the export or processing of antigens for MHC-I. Consistent with this, Mtb mutants lacking PknG or PtpB show increased macrophage activation and improved intracellular control, consistent with enhanced antigen availability for CD8^+^ T-cell priming (134, 157), presumably because normal antigen degradation and export are restored in the absence of these factors. Thus, in wild-type Mtb infection, PknG- and PtpB-mediated modifications to vesicle trafficking likely reduce the amount of bacterial peptides reaching the MHC-I pathway. It is telling that pathogens like Mtb and *Leishmania* – which rely heavily on evading Th1 CD4^+^ cells – also find ways to dampen CD8^+^ T-cell engagement. In chronic leishmaniasis, although CD8^+^ T cells are not the dominant mediators of parasite control, *Leishmania* still limits MHC-I antigen presentation. Persistent *Leishmania* infection is associated with an IL-10^high, IL-12/IL-15^low cytokine environment, which dampens antigen presentation and effector T-cell function, thereby promoting persistence (158). Additionally, by preventing effective CD4^+^ T-cell help, through MHC-II downregulation and IL-12 suppression as described, *Leishmania* indirectly impairs CD8^+^ T-cell priming and memory generation, since Th1 cytokines like IL-2 and IFN-γ from CD4^+^ cells are important for optimal CD8^+^ responses. Thus, intracellular parasites and bacteria converge on a two-pronged approach: disable MHC-II to weaken CD4^+^ T-cell help and interfere with MHC-I pathways to directly weaken CD8^+^ T-cell responses.

## Pathogen-encoded kinases and phosphatases: convergent evasion strategies

Underlying the above mechanisms are specific pathogen-encoded enzymes that directly modify host phosphorylation events. Mtb secretes multiple Ser/Thr kinases, such as PknG, and tyrosine phosphatases, such as PtpA and PtpB, that cumulatively reprogram host cell signaling. PtpB dephosphorylates components of the MAPK and NF-κB pathways, inhibits pro-inflammatory cytokine production, and pushes macrophages toward an anti-inflammatory, non-antigen-presenting state (157). Inhibition of Mtb’s phosphatases can reverse these effects, small-molecule inhibitors of PtpA and PtpB have been shown to restore normal macrophage activation and improve clearance of Mtb in infected models (157). Protozoan parasites likewise encode their own repertoires of secreted kinases and phosphatases that target host signaling. *T. gondii*’s rhoptry kinases include not only ROP18, which phosphorylates IRGs to protect the parasitophorous vacuole, but also ROP16, which phosphorylates STAT6 and STAT3 to skew the host cell toward an anti-inflammatory, M2-like phenotype with low IL-12 and reduced MHC expression. *Leishmania* parasites, for their part, deliver effectors that manipulate host phosphorylation cascades. Leishmania casein kinase 1 (L-CK1) and other secreted parasite kinases can enter the host cytosol and interfere with host protein phosphorylation circuits, such as those regulating NF-κB activation. More prominently, *Leishmania* uses GP63 not just to activate SHP-1, but also to cleave various host kinases, including PKC and MAPKs, effectively shutting off those signaling pathways. GP63’s ability to act as an immunomodulatory “pseudo-phosphatase”, by activating SHP-1 and inactivating kinases, closely parallels Mtb’s use of PtpA/PtpB, and in both cases, the pathogen sabotages host kinase signaling to paralyze antigen presentation. Diverse pathogens deploy specialized kinases and phosphatases or proteases that affect phosphorylation cascades to converge on the same endpoints of blocking antigen processing and presentation.

Across these diverse pathogens, a common logic emerges: phosphorylation is not only a host signaling tool, but a molecular vulnerability repeatedly exploited by intracellular microbes. By orchestrating these phosphoregulation-based disruptions, intracellular bacteria and parasites profoundly reduce the display of microbial antigens, including phosphopeptides, on MHC molecules, thereby blunting the activation of pathogen-specific T cells. This immune subversion is so crucial to pathogen success that hosts have evolved countermeasures; for example, even minor “leaks” in these evasion tactics, such as some phosphoantigens escaping degradation or certain bacterial peptides being cross-presented, can alert T cells and contribute to protective immunity. Understanding these parallel tricks used by bacteria and parasites not only sheds light on fundamental immune evasion mechanisms but also suggests potential therapeutic targets. Intervening at critical phosphorylation nodes with host-directed kinase inhibitors or pathogen-directed phosphatase inhibitors could broadly restore antigen presentation and T-cell recognition in these infections. In summary, phosphoregulation represents a recurring theme in immune evasion across pathogens, with intracellular microbes repeatedly targeting host signaling pathways to shut down antigen presentation and slip under the radar of adaptive immunity.

## Phosphorylation-dependent control of cytokine production and T-cell polarization

Beyond assembling the proximal signalosome, phosphorylation is the principal molecular switch that converts antigen recognition into defined cytokine programs. Engagement of the T-cell receptor (TCR) triggers Lck-mediated phosphorylation of CD3/ζ-chain ITAMs, recruitment and activation of ZAP-70, and phosphorylation of the adaptor LAT, which nucleates a complex that activates PLCγ1 and the downstream NFAT, NF-κB, and AP-1 transcription factors (159, 160). The phosphorylation-dependent, calcineurin-driven dephosphorylation and nuclear translocation of NFAT is especially decisive, as it licenses transcription of *IL2*, *IFNG*, and other effector cytokine loci (161). Thus, the strength, duration, and topology of these phosphorylation events set not only whether a T cell is activated but the quantitative and qualitative character of the cytokines it will secrete.

The differentiation of naïve CD4^+^ T cells into distinct effector lineages is then governed by a second phosphorylation layer: cytokine-receptor–coupled Janus kinase (JAK) activation and the tyrosine phosphorylation of signal transducer and activator of transcription (STAT) proteins (162, 163). Each polarizing cytokine milieu is transduced by a characteristic phospho-STAT that induces a lineage-defining transcription factor: IL-12/IFN-γ→STAT4/STAT1→T-bet for Th1 and IFN-γ output; IL-4→STAT6→GATA3 for Th2 and IL-4/IL-5/IL-13; IL-6 plus TGF-β→STAT3→RORγt for Th17 and IL-17; and IL-2→STAT5→FoxP3 for regulatory T cells (164) Because these master regulators reinforce their own cytokines while cross-inhibiting alternative programs, the balance and kinetics of STAT phosphorylation effectively encode the Th1/Th2/Th17/Treg decision, and even modest shifts in phospho-STAT dosage can redirect polarization and effector function.

**Figure 3:**
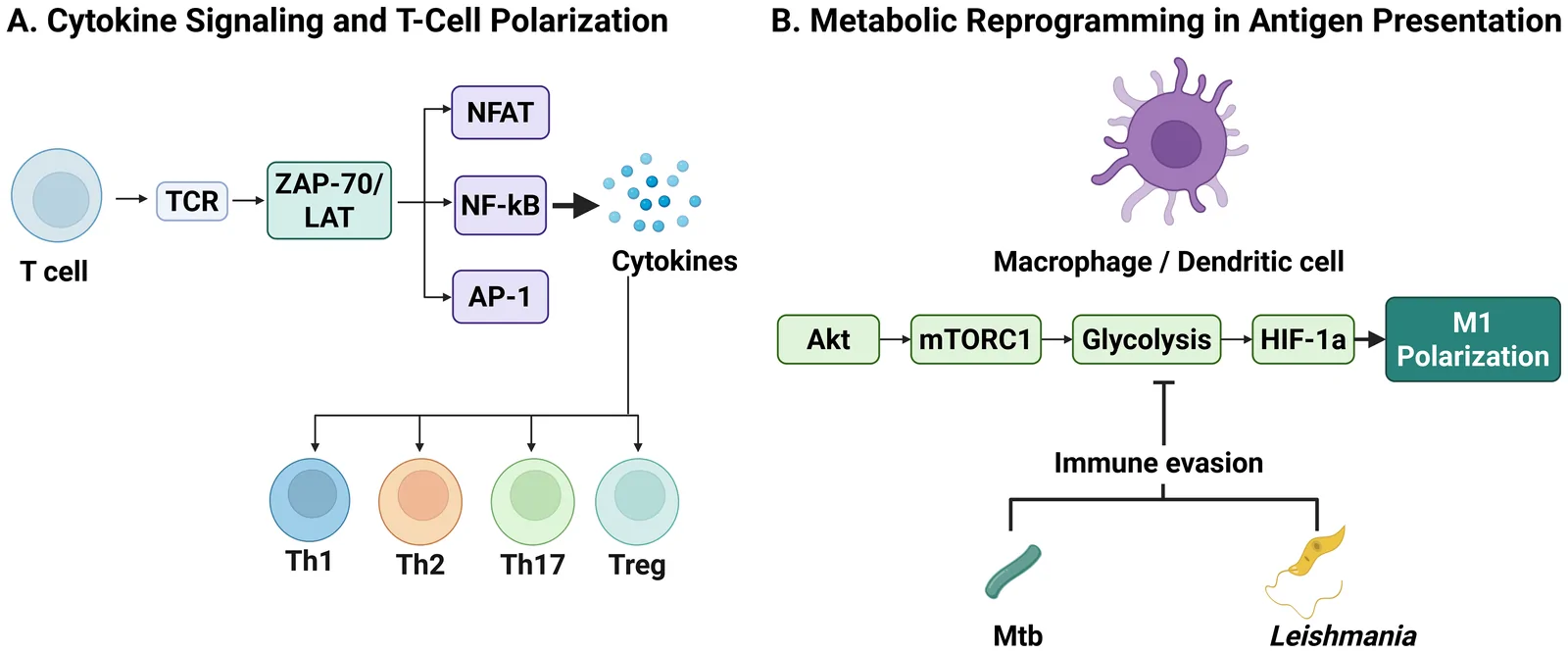
Phosphorylation-dependent regulation of cytokine signaling, T-cell polarization, and metabolic reprogramming in antigen presentation. **(A)** TCR engagement triggers ZAP-70/LAT-dependent phosphorylation cascades that activate NFAT, NF-κB, and AP-1, driving cytokine production. Cytokine-induced JAK-STAT signaling subsequently directs CD4+ T-cell differentiation into Th1, Th2, Th17, or Treg lineages. **(B)** In antigen-presenting cells, Akt-mTORC1 signaling drives a glycolytic shift that supports HIF-1α-dependent M1 macrophage polarization and effective antigen presentation. Intracellular pathogens such as *Mycobacterium tuberculosis* (Mtb) and *Leishmania* spp. subvert this metabolic program by suppressing host glycolytic flux, thereby impairing macrophage activation and enabling immune evasion.

These principles bear directly on the immunobiology of phosphopeptides. Phosphorylated peptides are naturally processed and presented by MHC class I and II molecules and are recognized by a dedicated repertoire of phosphopeptide-specific T cells, establishing the phosphorylation mark itself as an immunogenic determinant (6, 56). Consequently, phosphopeptides act at two intersecting levels of the pathways above: as antigens whose recognition initiates TCR-proximal phosphorylation cascades, and as immunogens whose presentation shapes the cytokine- and STAT-driven polarization of the responding cells. This dual role has practical implications for phosphopeptide-based vaccine and immunotherapy design, where the goal is not merely to elicit phosphopeptide-specific T cells but to bias their differentiation toward a protective effector profile—for example, a balanced Th1/Th2 response with robust IFN-γ and antigen-specific antibody. Framing phosphopeptides within this phosphorylation-to-polarization axis therefore clarifies how post-translational modification governs not only antigen identity but the downstream quality of T-cell immunity (**Figure 3**).

## Phosphorylation as a distinct immunological axis

Although multiple post-translational modifications (PTMs), including glycosylation, acetylation, citrullination, ubiquitination, and methylation, contribute to antigen diversification and immune recognition, phosphorylation possesses several unique immunological characteristics that distinguish it as a particularly dynamic source of antigenic plasticity. Unlike many other PTMs, phosphorylation is highly rapid, reversible, and tightly coupled to intracellular signaling cascades that regulate cellular activation, stress responses, oncogenic transformation, infection, and immune modulation (165, 166). As a result, phosphopeptides may provide real-time immunological snapshots of dysregulated cellular signaling states that are not directly captured by genomic mutations or constitutive protein expression alone (167). In contrast, glycosylation primarily influences protein folding, extracellular interactions, and steric shielding, whereas acetylation and methylation are more commonly associated with epigenetic and transcriptional regulation (168). Importantly, phosphorylation frequently occurs at solvent-exposed serine, threonine, and tyrosine residues capable of directly altering peptide–MHC binding affinity and T-cell receptor contact chemistry, thereby generating immunologically distinct neoepitopes (169, 170). Furthermore, aberrant kinase signaling is a hallmark of cancer, chronic infection, and inflammatory disease, providing a mechanistic link between phosphoproteome dysregulation and antigen presentation. Nevertheless, other PTMs also contribute substantially to immune recognition and may cooperate with phosphorylation to shape the broader landscape of modified antigen presentation (171–173).

## Conclusion and future perspectives

Phosphorylated antigens represent a distinct immunological category that integrates cell signaling with antigen identity. By altering peptide processing, MHC stability, and TCR engagement, phosphorylation reshapes immune recognition across cancer and infection. However, significant challenges remain. The identification of stably presented phosphopeptides *in vivo* is technically demanding, their dynamic and tissue-specific expression complicates biomarker development, and the risk of off-target T cell responses necessitates careful validation. Moving forward, integrating phosphoproteomics, immunopeptidomics, artificial intelligence-based epitope prediction, and high-dimensional immune profiling will be essential. These approaches will clarify which phospho-epitopes are stably presented *in vivo* and distinguish immunogenic targets from transient signaling by-products. Phosphorylation is not merely a regulatory modification; it is an immunological signal. Future work must define the phospho-immunome *in vivo* across infection and cancer to determine whether phosphorylation represents a universal axis of immune discrimination.

## References

[1] Qu Z Dong J Zhang ZY . Protein tyrosine phosphatases as emerging targets for cancer immunotherapy. Br J Pharmacol. (2026) 183:1233–49. doi: 10.1111/bph.16304 38116815 PMC11186978

[2] Zhou X Sun SC . Targeting ubiquitin signaling for cancer immunotherapy. Signal Transduct Target Ther. (2021) 6:16. doi: 10.1038/s41392-020-00421-2 33436547 PMC7804490

[3] Xie N Shen G Gao W Huang Z Huang C Fu L . Neoantigens: promising targets for cancer therapy. Signal Transduct Target Ther. (2023) 8:9. doi: 10.1038/s41392-022-01270-x 36604431 PMC9816309

[4] Mpakali A Stratikos E . The role of antigen processing and presentation in cancer and the efficacy of immune checkpoint inhibitor immunotherapy. Cancers (Basel). (2021) 13. doi: 10.3390/cancers13010134 33406696 PMC7796214

[5] Mohammed F Cobbold M Zarling AL Salim M Barrett-Wilt GA Shabanowitz J . Phosphorylation-dependent interaction between antigenic peptides and MHC class I: a molecular basis for the presentation of transformed self. Nat Immunol. (2008) 9:1236–43. doi: 10.1038/ni.1660 18836451 PMC2596764

[6] Zarling AL Ficarro SB White FM Shabanowitz J Hunt DF Engelhard VH . Phosphorylated peptides are naturally processed and presented by major histocompatibility complex class I molecules *in vivo*. J Exp Med. (2000) 192:1755–62. doi: 10.1084/jem.192.12.1755 11120772 PMC2213507

[7] Mahoney KE Reser L Ruiz Cuevas MV Abelin JG Shabanowitz J Hunt DF . Identification of post-translationally modified MHC class I-associated peptides as potential cancer immunotherapeutic targets. Mol Cell Proteomics. (2025) 24:100971. doi: 10.1016/j.mcpro.2025.100971 40239839 PMC12362676

[8] Depontieu FR Qian J Zarling AL McMiller TL Salay TM Norris A . Identification of tumor-associated, MHC class II-restricted phosphopeptides as targets for immunotherapy. Proc Natl Acad Sci USA. (2009) 106:12073–8. doi: 10.1073/pnas.0903852106 19581576 PMC2715484

[9] Mahoney KE Shabanowitz J Hunt DF . MHC phosphopeptides: promising targets for immunotherapy of cancer and other chronic diseases. Mol Cell Proteomics. (2021) 20:100112. doi: 10.1016/j.mcpro.2021.100112 34129940 PMC8724925

[10] Molvi Z Klatt MG Dao T Urraca J Scheinberg DA O'Reilly RJ . The landscape of MHC-presented phosphopeptides yields actionable shared tumor antigens for cancer immunotherapy across multiple HLA alleles. J Immunother Cancer. (2023) 11. doi: 10.1101/2023.02.08.527552 37775115 PMC10546156

[11] Petersen J Wurzbacher SJ Williamson NA Ramarathinam SH Reid HH Nair AK . Phosphorylated self-peptides alter human leukocyte antigen class I-restricted antigen presentation and generate tumor-specific epitopes. Proc Natl Acad Sci USA. (2009) 106:2776–81. doi: 10.1073/pnas.0812901106 19196958 PMC2650342

[12] Mata-Haro V Cekic C Martin M Chilton PM Casella CR Mitchell TC . The vaccine adjuvant monophosphoryl lipid A as a TRIF-biased agonist of TLR4. Science. (2007) 316:1628–32. doi: 10.1126/science.1138963 17569868

[13] Wang YQ Bazin-Lee H Evans JT Casella CR Mitchell TC . MPL adjuvant contains competitive antagonists of human TLR4. Front Immunol. (2020) 11:577823. doi: 10.3389/fimmu.2020.577823 33178204 PMC7596181

[14] Li D Wu M . Pattern recognition receptors in health and diseases. Signal Transduct Target Ther. (2021) 6:291. doi: 10.1038/s41392-021-00687-0 34344870 PMC8333067

[15] Brubaker SW Bonham KS Zanoni I Kagan JC . Innate immune pattern recognition: a cell biological perspective. Annu Rev Immunol. (2015) 33:257–90. doi: 10.1146/annurev-immunol-032414-112240 25581309 PMC5146691

[16] Watts BA George T Sherwood ER Good DW . Monophosphoryl lipid A induces protection against LPS in medullary thick ascending limb through a TLR4-TRIF-PI3K signaling pathway. Am J Physiol Renal Physiol. (2017) 313:F103–15. doi: 10.1152/ajprenal.00064.2017 28356284 PMC5538844

[17] Stark R Choi H Koch S Lamb F Sherwood E . Monophosphoryl lipid A inhibits the cytokine response of endothelial cells challenged with LPS. Innate Immun. (2015) 21:565–74. doi: 10.1177/1753425914564172 25540284 PMC4480205

[18] Carter D De La Rosa G Garcon N Moon HM Nam HJ Skibinski DAG . The success of toll-like receptor 4 based vaccine adjuvants. Vaccine. (2025) 61:127413. doi: 10.1016/j.vaccine.2025.127413 40570747

[19] Monie A Hung CF Roden R Wu TC . Cervarix: a vaccine for the prevention of HPV 16, 18-associated cervical cancer. Biologics. (2008) 2:97–105. doi: 10.4147/hto-080300 PMC2727782 19707432

[20] Zhao T Cai Y Jiang Y He X Wei Y Yu Y . Vaccine adjuvants: mechanisms and platforms. Signal Transduct Target Ther. (2023) 8:283. doi: 10.1038/s41392-023-01557-7 37468460 PMC10356842

[21] Scholz C Tampe R . The peptide-loading complex--antigen translocation and MHC class I loading. Biol Chem. (2009) 390:783–94. doi: 10.1515/BC.2009.069 19426129

[22] Li Y Salter-Cid L Vitiello A Preckel T Lee JD Angulo A . Regulation of transporter associated with antigen processing by phosphorylation. J Biol Chem. (2000) 275:24130–5. doi: 10.1074/jbc.m003617200 10823836

[23] Guo X Wang X Wang Z Banerjee S Yang J Huang L . Site-specific proteasome phosphorylation controls cell proliferation and tumorigenesis. Nat Cell Biol. (2016) 18:202–12. doi: 10.1038/ncb3289 26655835 PMC4844191

[24] Turowec JP Zukowski SA Knight JD Smalley DM Graves LM Johnson GL . An unbiased proteomic screen reveals caspase cleavage is positively and negatively regulated by substrate phosphorylation. Mol Cell Proteomics. (2014) 13:1184–97. doi: 10.1074/mcp.m113.037374 24556848 PMC4014278

[25] Sijts EJ Kloetzel PM . The role of the proteasome in the generation of MHC class I ligands and immune responses. Cell Mol Life Sci. (2011) 68:1491–502. doi: 10.1007/s00018-011-0657-y 21387144 PMC3071949

[26] Kruger E Kuckelkorn U Sijts A Kloetzel PM . The components of the proteasome system and their role in MHC class I antigen processing. Rev Physiol Biochem Pharmacol. (2003) 148:81–104. doi: 10.1007/s10254-003-0010-4 12687403

[27] Wu C Ba Q Lu D Li W Salovska B Hou P . Global and site-specific effect of phosphorylation on protein turnover. Dev Cell. (2021) 56:111–24. doi: 10.1016/j.devcel.2020.10.025 33238149 PMC7855865

[28] Andersen MH Bonfill JE Neisig A Arsequell G Sondergaard I Valencia G . Phosphorylated peptides can be transported by TAP molecules, presented by class I MHC molecules, and recognized by phosphopeptide-specific CTL. J Immunol. (1999) 163:3812–8. doi: 10.4049/jimmunol.163.7.3812 10490979

[29] Hewitt EW . The MHC class I antigen presentation pathway: strategies for viral immune evasion. Immunology. (2003) 110:163–9. doi: 10.1046/j.1365-2567.2003.01738.x 14511229 PMC1783040

[30] DhatChinamoorthy K Colbert JD Rock KL . Cancer immune evasion through loss of MHC class I antigen presentation. Front Immunol. (2021) 12:636568. doi: 10.3389/fimmu.2021.636568 33767702 PMC7986854

[31] Rana PS Ignatz-Hoover JJ Guo C Mosley AL Malek E Federov Y . Immunoproteasome activation expands the MHC class I immunopeptidome, unmasks neoantigens, and enhances T-cell anti-myeloma activity. Mol Cancer Ther. (2024) 23:1743–60. doi: 10.1158/1535-7163.mct-23-0931 39210605 PMC11612626

[32] Grotzke JE Sengupta D Lu Q Cresswell P . The ongoing saga of the mechanism(s) of MHC class I-restricted cross-presentation. Curr Opin Immunol. (2017) 46:89–96. doi: 10.1016/j.coi.2017.03.015 28528219 PMC5554740

[33] Zhao Y Sun M Zhang N Liu X Yue C Feng L . Phosphosite-dependent presentation of dual phosphorylated peptides by MHC class I molecules. iScience. (2022) 25:104013. doi: 10.1016/j.isci.2022.104013 35310951 PMC8931367

[34] Sandalova T Sala BM Achour A . Structural aspects of chemical modifications in the MHC-restricted immunopeptidome; implications for immune recognition. Front Chem. (2022) 10:861609. doi: 10.3389/fchem.2022.861609 36017166 PMC9395651

[35] Li Y Depontieu FR Sidney J Salay TM Engelhard VH Hunt DF . Structural basis for the presentation of tumor-associated MHC class II-restricted phosphopeptides to CD4+ T cells. J Mol Biol. (2010) 399:596–603. doi: 10.1016/j.jmb.2010.04.037 20417641 PMC2904831

[36] Patskovsky Y Natarajan A Patskovska L Nyovanie S Joshi B Morin B . Molecular mechanism of phosphopeptide neoantigen immunogenicity. Nat Commun. (2023) 14:3763. doi: 10.21203/rs.3.rs-2327641/v1 37353482 PMC10290117

[37] Marcilla M . Immunopeptidomic analysis of the phosphopeptidome displayed by HLA class I molecules. Methods Mol Biol. (2022) 2420:149–58. doi: 10.1007/978-1-0716-1936-0_12 34905172

[38] Apoorvi T Yury P Iryna V Michelle K . Phosphopeptide neoantigens as emerging targets in cancer immunotherapy. J Cancer Immunol (Wilmington). (2024) 6:135–47. doi: 10.33696/cancerimmunol.6.094 39713021 PMC11661817

[39] Hao Q Long Y Yang Y Deng Y Ding Z Yang L . Development and clinical applications of therapeutic cancer vaccines with individualized and shared neoantigens. Vaccines (Basel). (2024) 12. doi: 10.3390/vaccines12070717 39066355 PMC11281709

[40] Engelhard VH Obeng RC Cummings KL Petroni GR Ambakhutwala AL Chianese-Bullock KA . MHC-restricted phosphopeptide antigens: preclinical validation and first-in-humans clinical trial in participants with high-risk melanoma. J Immunother Cancer. (2020) 8. doi: 10.1136/jitc-2019-000262 32385144 PMC7228659

[41] Dao T Mun SS Molvi Z Korontsvit T Klatt MG Khan AG . A TCR mimic monoclonal antibody reactive with the "public" phospho-neoantigen pIRS2/HLA-A*02:01 complex. JCI Insight. (2022) 7. doi: 10.1172/jci.insight.151624 35260532 PMC8983142

[42] Mariuzza RA Agnihotri P Orban J . The structural basis of T-cell receptor (TCR) activation: an enduring enigma. J Biol Chem. (2020) 295:914–25. doi: 10.1016/s0021-9258(17)49904-2 PMC6983839 31848223

[43] Petrova G Ferrante A Gorski J . Cross-reactivity of T cells and its role in the immune system. Crit Rev Immunol. (2012) 32:349–72. doi: 10.1615/critrevimmunol.v32.i4.50 23237510 PMC3595599

[44] Teague RM Greenberg PD Fowler C Huang MZ Tan X Morimoto J . Peripheral CD8+ T cell tolerance to self-proteins is regulated proximally at the T cell receptor. Immunity. (2008) 28:662–74. doi: 10.1016/j.immuni.2008.03.012 18424189 PMC3443683

[45] Yao Z Zeng Y Liu C Jin H Wang H Zhang Y . Focusing on CD8(+) T-cell phenotypes: improving solid tumor therapy. J Exp Clin Cancer Res. (2024) 43:266. doi: 10.1186/s13046-024-03195-5 39342365 PMC11437975

[46] Xing Y Hogquist KA . T-cell tolerance: central and peripheral. Cold Spring Harb Perspect Biol. (2012) 4. doi: 10.1101/cshperspect.a006957 22661634 PMC3367546

[47] Singh NK Riley TP Baker SCB Borrman T Weng Z Baker BM . Emerging concepts in TCR specificity: rationalizing and (maybe) predicting outcomes. J Immunol. (2017) 199:2203–13. doi: 10.4049/jimmunol.1700744 28923982 PMC5679125

[48] Szeto C Lobos CA Nguyen AT Gras S . TCR recognition of peptide-MHC-I: rule makers and breakers. Int J Mol Sci. (2020) 22. doi: 10.3390/ijms22010068 33374673 PMC7793522

[49] Aparicio B Theunissen P Hervas-Stubbs S Fortes P Sarobe P . Relevance of mutation-derived neoantigens and non-classical antigens for anticancer therapies. Hum Vaccin Immunother. (2024) 20:2303799. doi: 10.1080/21645515.2024.2303799 38346926 PMC10863374

[50] Lang F Schrors B Lower M Tureci O Sahin U . Identification of neoantigens for individualized therapeutic cancer vaccines. Nat Rev Drug Discov. (2022) 21:261–82. doi: 10.1038/s41573-021-00387-y 35105974 PMC7612664

[51] Chen B Zhu H Yang B Cao J . The dichotomous role of immunoproteasome in cancer: Friend or foe? Acta Pharm Sin B. (2023) 13:1976–89. doi: 10.1016/j.apsb.2022.11.005 37250147 PMC10213805

[52] Rana PS Ignatz-Hoover JJ Driscoll JJ . Targeting proteasomes and the MHC class I antigen presentation machinery to treat cancer, infections and age-related diseases. Cancers (Basel). (2023) 15. doi: 10.3390/cancers15235632 38067336 PMC10705104

[53] Lange T Kasper L Gresnigt MS Brunke S Hube B . Under pressure" - How fungi evade, exploit, and modulate cells of the innate immune system. Semin Immunol. (2023) 66:101738. doi: 10.1016/j.smim.2023.101738 PMC10109127 36878023

[54] Santambrogio L Clement C Osan J Becerra A Mota I Nanaware P . Molecular mimicry in pathogen immune evasion. J Immunol. (2024) 212:1438–4569. doi: 10.4049/jimmunol.212.supp.1438.4569

[55] Suliman BA . Potential clinical implications of molecular mimicry-induced autoimmunity. Immun Inflammation Dis. (2024) 12:e1178. doi: 10.1002/iid3.1178 38415936 PMC10832321

[56] Cobbold M De La Pena H Norris A Polefrone JM Qian J English AM . MHC class I-associated phosphopeptides are the targets of memory-like immunity in leukemia. Sci Transl Med. (2013) 5:203ra125. doi: 10.1126/scitranslmed.3006061 24048523 PMC4071620

[57] Chattopadhyay S Sen GC . Tyrosine phosphorylation in toll-like receptor signaling. Cytokine Growth Factor Rev. (2014) 25:533–41. doi: 10.1016/j.cytogfr.2014.06.002 25022196 PMC4254339

[58] Ciesielska A Matyjek M Kwiatkowska K . TLR4 and CD14 trafficking and its influence on LPS-induced pro-inflammatory signaling. Cell Mol Life Sci. (2021) 78:1233–61. doi: 10.1007/s00018-020-03656-y 33057840 PMC7904555

[59] Kawasaki T Kawai T . Toll-like receptor signaling pathways. Front Immunol. (2014) 5:461. doi: 10.3389/fimmu.2014.00461 25309543 PMC4174766

[60] Brown J Wang H Hajishengallis GN Martin M . TLR-signaling networks: An integration of adaptor molecules, kinases, and cross-talk. J Dent Res. (2011) 90:417–27. doi: 10.1177/0022034510381264 PMC3075579 20940366

[61] Weintz G Olsen JV Fruhauf K Niedzielska M Amit I Jantsch J . The phosphoproteome of toll-like receptor-activated macrophages. Mol Syst Biol. (2010) 6:371. doi: 10.1038/msb.2010.29 20531401 PMC2913394

[62] Lannoy V Cote-Biron A Asselin C Rivard N . Phosphatases in toll-like receptors signaling: The unfairly-forgotten. Cell Commun Signal. (2021) 19:10. doi: 10.1186/s12964-020-00693-9 33494775 PMC7829650

[63] Popli S Chakravarty S Fan S Glanz A Aras S Nagy LE . IRF3 inhibits nuclear translocation of NF-kappaB to prevent viral inflammation. Proc Natl Acad Sci USA. (2022) 119:e2121385119. doi: 10.1073/pnas.2121385119 36067309 PMC9478676

[64] Carenza C Calcaterra F Oriolo F Di Vito C Ubezio M Della Porta MG . Costimulatory molecules and immune checkpoints are differentially expressed on different subsets of dendritic cells. Front Immunol. (2019) 10:1325. doi: 10.3389/fimmu.2019.01325 31244860 PMC6579930

[65] Ho NI Huis In 't Veld LGM Raaijmakers TK Adema GJ . Adjuvants enhancing cross-presentation by dendritic cells: The key to more effective vaccines? Front Immunol. (2018) 9:2874. doi: 10.3389/fimmu.2018.02874 30619259 PMC6300500

[66] Ma X Yan W Zheng H Du Q Zhang L Ban Y . Regulation of IL-10 and IL-12 production and function in macrophages and dendritic cells. F1000Res. (2015) 4. doi: 10.12688/f1000research.7010.1 26918147 PMC4754024

[67] Ross SH Rollings C Anderson KE Hawkins PT Stephens LR Cantrell DA . Phosphoproteomic analyses of interleukin 2 signaling reveal integrated JAK kinase-dependent and -independent networks in CD8(+) T cells. Immunity. (2016) 45:685–700. doi: 10.1016/j.immuni.2016.07.022 27566939 PMC5040828

[68] Ardito F Giuliani M Perrone D Troiano G Lo Muzio L . The crucial role of protein phosphorylation in cell signaling and its use as targeted therapy (review). Int J Mol Med. (2017) 40:271–80. doi: 10.3892/ijmm.2017.3036 28656226 PMC5500920

[69] Adiko AC Babdor J Gutierrez-Martinez E Guermonprez P Saveanu L . Intracellular transport routes for MHC I and their relevance for antigen cross-presentation. Front Immunol. (2015) 6:335. doi: 10.3389/fimmu.2015.00335 26191062 PMC4489332

[70] Li X You J Hong L Liu W Guo P Hao X . Neoantigen cancer vaccines: A new star on the horizon. Cancer Biol Med. (2023) 21:274–311. doi: 10.20892/j.issn.2095-3941.2023.0395 38164734 PMC11033713

[71] Shah BA Holden JA Lenzo JC Hadjigol S O'Brien-Simpson NM . Multi-disciplinary approaches paving the way for clinically effective peptide vaccines for cancer. NPJ Vaccines. (2025) 10:68. doi: 10.1038/s41541-025-01118-9 40204832 PMC11982186

[72] Xie N Shen G Huang C Zhu H . Neoantigen-driven personalized tumor therapy: An update from discovery to clinical application. Chin Med J (Engl). (2025) 138:2057–90. doi: 10.1097/cm9.0000000000003708 40757404 PMC12407177

[73] Moon DO . Deciphering the role of BCAR3 in cancer progression: Gene regulation, signal transduction, and therapeutic implications. Cancers (Basel). (2024) 16. doi: 10.3390/cancers16091674 38730626 PMC11083344

[74] Zarling AL Obeng RC Desch AN Pinczewski J Cummings KL Deacon DH . MHC-restricted phosphopeptides from insulin receptor substrate-2 and CDC25b offer broad-based immunotherapeutic agents for cancer. Cancer Res. (2014) 74:6784–95. doi: 10.1158/0008-5472.can-14-0043 25297629 PMC4252710

[75] Melssen MM Petroni GR Chianese-Bullock KA Wages NA Grosh WW Varhegyi N . A multipeptide vaccine plus toll-like receptor agonists LPS or polyICLC in combination with incomplete Freund's adjuvant in melanoma patients. J Immunother Cancer. (2019) 7:163. doi: 10.1186/s40425-019-0625-x 31248461 PMC6598303

[76] Slingluff JCL . Evaluation of a multi-phosphopeptide vaccine plus polyICLC in participants with high risk and advanced Malignancies (Mel59) (2016). Available online at: https://clinicaltrials.gov/study/NCT01846143 (Assessed July 19, 2026).

[77] Wu X Liu YK Iliuk AB Tao WA . Mass spectrometry-based phosphoproteomics in clinical applications. Trends Analyt Chem. (2023) 163. doi: 10.1016/j.trac.2023.117066 37215489 PMC10195102

[78] Mangalaparthi KK Madugundu AK Ryan ZC Garapati K Peterson JA Dey G . Digging deeper into the immunopeptidome: Characterization of post-translationally modified peptides presented by MHC I. J Proteins Proteom. (2021) 12:151–60. doi: 10.1007/s42485-021-00066-x 36619276 PMC9807509

[79] Chrusciel E Urban-Wojciuk Z Arcimowicz L Kurkowiak M Kowalski J Gliwinski M . Adoptive cell therapy-harnessing antigen-specific T cells to target solid tumours. Cancers (Basel). (2020) 12. doi: 10.3390/cancers12030683 PMC7140076 32183246

[80] Eslami M Fadaee Dowlat B Yaghmayee S Habibian A Keshavarzi S Oksenych V . Next-generation vaccine platforms: Integrating synthetic biology, nanotechnology, and systems immunology for improved immunogenicity. Vaccines (Basel). (2025) 13. doi: 10.3390/vaccines13060588 40573919 PMC12197771

[81] Lancaster NM Sinitcyn P Forny P Peters-Clarke TM Fecher C Smith AJ . Fast and deep phosphoproteome analysis with the Orbitrap Astral mass spectrometer. Nat Commun. (2024) 15:7016. doi: 10.1038/s41467-024-51274-0 39147754 PMC11327265

[82] Gerritsen JS White FM . Phosphoproteomics: A valuable tool for uncovering molecular signaling in cancer cells. Expert Rev Proteomics. (2021) 18:661–74. doi: 10.1080/14789450.2021.1976152 34468274 PMC8628306

[83] Gnjatic S Bronte V Brunet LR Butler MO Disis ML Galon J . Identifying baseline immune-related biomarkers to predict clinical outcome of immunotherapy. J Immunother Cancer. (2017) 5:44. doi: 10.1186/s40425-017-0243-4 28515944 PMC5432988

[84] Ramroop JR Stein MN Drake JM . Impact of phosphoproteomics in the era of precision medicine for prostate cancer. Front Oncol. (2018) 8:28. doi: 10.3389/fonc.2018.00028 29503809 PMC5820335

[85] Rath JA Arber C . Engineering strategies to enhance TCR-based adoptive T cell therapy. Cells. (2020) 9. doi: 10.3390/cells9061485 32570906 PMC7349724

[86] Ecsedi M McAfee MS Chapuis AG . The anticancer potential of T cell receptor-engineered T cells. Trends Cancer. (2021) 7:48–56. doi: 10.1016/j.trecan.2020.09.002 32988787 PMC7770096

[87] Kim CG Sang YB Lee JH Chon HJ . Combining cancer vaccines with immunotherapy: Establishing a new immunological approach. Int J Mol Sci. (2021) 22. doi: 10.3390/ijms22158035 34360800 PMC8348347

[88] Pearson H Daouda T Granados DP Durette C Bonneil E Courcelles M . MHC class I-associated peptides derive from selective regions of the human genome. J Clin Invest. (2016) 126:4690–701. doi: 10.1172/jci88590 27841757 PMC5127664

[89] Newkirk SE Kelly JJ Hourn N Bhandari S Spencer N Pires MM . Mapping the modification landscape of MHC-I epitopes: A framework for immunogenic peptidomimetic antigen design. bioRxiv. (2026). doi: 10.64898/2026.03.06.710184 42191216 PMC13288458

[90] Solleder M Guillaume P Racle J Michaux J Pak HS Muller M . Mass spectrometry based immunopeptidomics leads to robust predictions of phosphorylated HLA class I ligands. Mol Cell Proteomics. (2020) 19:390–404. doi: 10.1074/mcp.tir119.001641 31848261 PMC7000122

[91] Zhao X Sankaran S Yap J Too CT Ho ZZ Dolton G . Nonstimulatory peptide-MHC enhances human T-cell antigen-specific responses by amplifying proximal TCR signaling. Nat Commun. (2018) 9:2716. doi: 10.1038/s41467-018-05288-0 30006605 PMC6045629

[92] Sturm T Sautter B Worner TP Stevanovic S Rammensee HG Planz O . Mild acid elution and MHC immunoaffinity chromatography reveal similar albeit not identical profiles of the HLA class I immunopeptidome. J Proteome Res. (2021) 20:289–304. doi: 10.1021/acs.jproteome.0c00386 33141586 PMC7786382

[93] Flender D Vilenne F Adams C Boonen K Valkenborg D Baggerman G . Exploring the dynamic landscape of immunopeptidomics: Unravelling posttranslational modifications and navigating bioinformatics terrain. Mass Spectrom Rev. (2025) 44:599–629. doi: 10.1002/mas.21905 39152539 PMC12147530

[94] Muneer G Chen CS Chen YJ . Advancements in global phosphoproteomics profiling: Overcoming challenges in sensitivity and quantification. Proteomics. (2025) 25:e202400087. doi: 10.1002/pmic.202400087 39696887 PMC11735659

[95] Stopfer LE Conage-Pough JE White FM . Quantitative consequences of protein carriers in immunopeptidomics and tyrosine phosphorylation MS(2) analyses. Mol Cell Proteomics. (2021) 20:100104. doi: 10.1016/j.mcpro.2021.100104 34052394 PMC8240026

[96] Gafken PR Lampe PD . Methodologies for characterizing phosphoproteins by mass spectrometry. Cell Commun Adhes. (2006) 13:249–62. doi: 10.1080/15419060601077917 17162667 PMC2185548

[97] von Stechow L Francavilla C Olsen JV . Recent findings and technological advances in phosphoproteomics for cells and tissues. Expert Rev Proteomics. (2015) 12:469–87. doi: 10.1586/14789450.2015.1078730 26400465 PMC4819829

[98] Riley NM Coon JJ . Phosphoproteomics in the age of rapid and deep proteome profiling. Anal Chem. (2016) 88:74–94. doi: 10.1021/acs.analchem.5b04123 26539879 PMC4790442

[99] Fila J Honys D . Enrichment techniques employed in phosphoproteomics. Amino Acids. (2012) 43:1025–47. doi: 10.1007/s00726-011-1111-z PMC3418503 22002794

[100] Li J Wang J Yan Y Li N Qing X Tuerxun A . Comprehensive evaluation of different TiO(2)-based phosphopeptide enrichment and fractionation methods for phosphoproteomics. Cells. (2022) 11. doi: 10.3390/cells11132047 35805136 PMC9265536

[101] Zhou H Low TY Hennrich ML van der Toorn H Schwend T Zou H . Enhancing the identification of phosphopeptides from putative basophilic kinase substrates using Ti (IV) based IMAC enrichment. Mol Cell Proteomics. (2011) 10:M110 006452. doi: 10.1074/mcp.a110.006452 21715320 PMC3205857

[102] Juanes-Velasco P Arias-Hidalgo C Garcia-Vaquero ML Sotolongo-Ravelo J Paino T Lecrevisse Q . Crucial parameters for immunopeptidome characterization: A systematic evaluation. Int J Mol Sci. (2024) 25. doi: 10.3390/ijms25179564 39273511 PMC11395153

[103] ElAbd H Bacher P Tholey A Lenz TL Franke A . Challenges and opportunities in analyzing and modeling peptide presentation by HLA-II proteins. Front Immunol. (2023) 14:1107266. doi: 10.3389/fimmu.2023.1107266 37063883 PMC10090296

[104] Abelin JG Cox AL . Innovations toward immunopeptidomics. Mol Cell Proteomics. (2024) 23:100823. doi: 10.1016/j.mcpro.2024.100823 39095021 PMC11419911

[105] Gfeller D Liu Y Racle J . Contemplating immunopeptidomes to better predict them. Semin Immunol. (2023) 66:101708. doi: 10.1016/j.smim.2022.101708 36621290

[106] Arshad S Cameron B Joglekar AV . Immunopeptidomics for autoimmunity: Unlocking the chamber of immune secrets. NPJ Syst Biol Appl. (2025) 11:10. doi: 10.1038/s41540-024-00482-x 39833247 PMC11747513

[107] Mohammed F Stones DH Zarling AL Willcox CR Shabanowitz J Cummings KL . The antigenic identity of human class I MHC phosphopeptides is critically dependent upon phosphorylation status. Oncotarget. (2017) 8:54160–72. doi: 10.18632/oncotarget.16952 28903331 PMC5589570

[108] Oseni SO Wang Y Hwu P . Advancing human leukocyte antigen-based cancer immunotherapy: From personalized to broad-spectrum strategies for genetically heterogeneous populations. Trends Cancer. (2025) 11:934–65. doi: 10.1016/j.trecan.2025.08.013 41046167 PMC12591376

[109] Alban TJ Riaz N Parthasarathy P Makarov V Kendall S Yoo SK . Neoantigen immunogenicity landscapes and evolution of tumor ecosystems during immunotherapy with nivolumab. Nat Med. (2024) 30:3209–22. doi: 10.1038/s41591-024-03240-y 39349627 PMC12066197

[110] Okada M Yamasaki S Sato GR Shimizu K Fujii SI . Neoantigen targeting as a novel approach for therapy-resistant tumors. Mol Diagn Ther. (2026) 30:495–514. doi: 10.1007/s40291-026-00842-9 41912995 PMC13172014

[111] Gillette MA Satpathy S Cao S Dhanasekaran SM Vasaikar SV Krug K . Proteogenomic characterization reveals therapeutic vulnerabilities in lung adenocarcinoma. Cell. (2020) 182:200–25.e235. doi: 10.1016/j.jtho.2019.12.031 32649874 PMC7373300

[112] Wang Y Olsen LK Jiao F Wang C Jiang KX Dou Y . A 15-layer multi-omics analysis of gastric cancer ecotypes provides therapeutic insights. Cell Rep Med. (2026) 7:102756. doi: 10.1016/j.xcrm.2026.102756 42013851 PMC13198309

[113] Bouhaddou M Memon D Meyer B White KM Rezelj VV Correa Marrero M . The global phosphorylation landscape of SARS-CoV-2 infection. Cell. (2020) 182:685–712.e619. doi: 10.1016/j.cell.2020.06.034 32645325 PMC7321036

[114] Wang L Zhang H Jiang H . Phosphorylation modifications and their role in viral pneumonia: Mechanisms and therapeutic implications. Infect Drug Resist. (2025) 18:6915–33. doi: 10.2147/idr.s562997 41476798 PMC12747894

[115] Lim Z Mohd-Ismail NKB Png E Sze CW Lin Q Hong W . Phosphoproteomics unravel HBV triggered rewiring of host phosphosignaling events. Int J Mol Sci. (2022) 23. doi: 10.3390/ijms23095127 35563518 PMC9104152

[116] Haro I Sanmarti R Gomara MJ . Implications of post-translational modifications in autoimmunity with emphasis on citrullination, homocitrullination and acetylation for the pathogenesis, diagnosis and prognosis of rheumatoid arthritis. Int J Mol Sci. (2022) 23. doi: 10.3390/ijms232415803 36555449 PMC9781636

[117] Zhai Y Wu K Lin Q Cao Z Jia Y Zhu P . Post-translational modified neoantigens in autoimmune diseases: Challenges of immune tolerance. Adv Sci (Weinh). (2025) 12:e01766. doi: 10.1002/advs.202501766 40538197 PMC12442617

[118] Safa A Vruzhaj I Gambirasi M Toffoli G . Antigenic dark matter: Unexplored post-translational modifications of tumor-associated and tumor-specific antigens in pancreatic cancer. Cancers (Basel). (2025) 17. doi: 10.3390/cancers17213506 41228299 PMC12609290

[119] Yewdell JW Balakrishnan A Winiarek G Holowka O Godlewski J Bronisz A . MHC class I immunopeptidome: Past, present, and future. Mol Cell Proteomics. (2022) 21:100230. doi: 10.1016/j.mcpro.2022.100230 35395404 PMC9243166

[120] Balakrishnan A Winiarek G Holowka O Godlewski J Bronisz A . Unlocking the secrets of the immunopeptidome: MHC molecules, ncRNA peptides, and vesicles in immune response. Front Immunol. (2025) 16:1540431. doi: 10.3389/fimmu.2025.1540431 39944685 PMC11814183

[121] Stopfer LE Mesfin JM Joughin BA Lauffenburger DA White FM . Multiplexed relative and absolute quantitative immunopeptidomics reveals MHC I repertoire alterations induced by CDK4/6 inhibition. Nat Commun. (2020) 11:2760. doi: 10.1038/s41467-020-16588-9 32488085 PMC7265461

[122] Pu T Peddle A Zhu J Tejpar S Verbandt S . Neoantigen identification: Technological advances and challenges. Methods Cell Biol. (2024) 183:265–302. doi: 10.1016/bs.mcb.2023.06.005 38548414

[123] Rasmussen M Fenoy E Harndahl M Kristensen AB Nielsen IK Nielsen M . Pan-specific prediction of peptide-MHC class I complex stability, a correlate of T cell immunogenicity. J Immunol. (2016) 197:1517–24. doi: 10.4049/jimmunol.1600582 27402703 PMC4976001

[124] Baumgartner CK Ferrante A Nagaoka M Gorski J Malherbe LP . Peptide-MHC class II complex stability governs CD4 T cell clonal selection. J Immunol. (2010) 184:573–81. doi: 10.4049/jimmunol.0902107 20007533 PMC2975033

[125] Liu YY Wang RJ Ru SS Gao F Liu W Zhang X . Comparative analysis of phosphorylated proteomes between plerocercoid and adult Spirometra mansoni reveals phosphoproteomic profiles of the medical tapeworm. Parasit Vectors. (2024) 17:371. doi: 10.1186/s13071-024-06454-8 39217359 PMC11366163

[126] Alam MM Solyakov L Bottrill AR Flueck C Siddiqui FA Singh S . Phosphoproteomics reveals malaria parasite protein kinase G as a signalling hub regulating egress and invasion. Nat Commun. (2015) 6:7285. doi: 10.1038/ncomms8285 26149123 PMC4507021

[127] Biscari L Maza MC Farre C Kaufman CD Amigorena S Fresno M . Sec22b-dependent antigen cross-presentation is a significant contributor of T cell priming during infection with the parasite Trypanosoma cruzi. Front Cell Dev Biol. (2023) 11:1138571. doi: 10.3389/fcell.2023.1138571 36936692 PMC10014565

[128] Feliu V Vasseur V Grover HS Chu HH Brown MJ Wang J . Location of the CD8 T cell epitope within the antigenic precursor determines immunogenicity and protection against the Toxoplasma gondii parasite. PloS Pathog. (2013) 9:e1003449. doi: 10.1371/journal.ppat.1003449 23818852 PMC3688528

[129] Sundar S Singh B . Understanding Leishmania parasites through proteomics and implications for the clinic. Expert Rev Proteomics. (2018) 15:371–90. doi: 10.1080/14789450.2018.1468754 PMC5970101 29717934

[130] Park BS Song DH Kim HM Choi BS Lee H Lee JO . The structural basis of lipopolysaccharide recognition by the TLR4-MD-2 complex. Nature. (2009) 458:1191–5. doi: 10.1038/nature07830 19252480

[131] Crotzer VL Blum JS . Autophagy and its role in MHC-mediated antigen presentation. J Immunol. (2009) 182:3335–41. doi: 10.4049/jimmunol.0803458 19265109 PMC2730830

[132] Shintre SS Dingli F Meyerhöfer N Gorgette O Thouvenot C Blumenthal DB . Dynamic phosphoproteomics and proteomics uncover Leishmania donovani-driven ferritin hijacking, contributing to the control of iron homeostasis and iron-related oxidative stress. bioRxiv. (2026). doi: 10.64898/2026.02.25.707907

[133] Jayachandran R Sundaramurthy V Combaluzier B Mueller P Korf H Huygen K . Survival of mycobacteria in macrophages is mediated by coronin 1-dependent activation of calcineurin. Cell. (2007) 130:37–50. doi: 10.1016/j.cell.2007.04.043 17632055

[134] Pradhan G Shrivastva R Mukhopadhyay S . Mycobacterial PknG targets the Rab7l1 signaling pathway to inhibit phagosome-lysosome fusion. J Immunol. (2018) 201:1421–33. doi: 10.4049/jimmunol.1800530 30037848

[135] Vergne I Chua J Lee HH Lucas M Belisle J Deretic V . Mechanism of phagolysosome biogenesis block by viable Mycobacterium tuberculosis. Proc Natl Acad Sci USA. (2005) 102:4033–8. doi: 10.1073/pnas.0409716102 15753315 PMC554822

[136] Wong D Bach H Sun J Hmama Z Av-Gay Y . Mycobacterium tuberculosis protein tyrosine phosphatase (PtpA) excludes host vacuolar-H+-ATPase to inhibit phagosome acidification. Proc Natl Acad Sci USA. (2011) 108:19371–6. doi: 10.1073/pnas.1109201108 22087003 PMC3228452

[137] Holm A Tejle K Magnusson KE Descoteaux A Rasmusson B . Leishmania donovani lipophosphoglycan causes periphagosomal actin accumulation: Correlation with impaired translocation of PKCalpha and defective phagosome maturation. Cell Microbiol. (2001) 3:439–47. doi: 10.1046/j.1462-5822.2001.00127.x 11437830

[138] Fentress SJ Behnke MS Dunay IR Mashayekhi M Rommereim LM Fox BA . Phosphorylation of immunity-related GTPases by a Toxoplasma gondii-secreted kinase promotes macrophage survival and virulence. Cell Host Microbe. (2010) 8:484–95. doi: 10.1016/j.chom.2010.11.005 21147463 PMC3013631

[139] Redford PS Murray PJ O'Garra A . The role of IL-10 in immune regulation during M. tuberculosis infection. Mucosal Immunol. (2011) 4:261–70. doi: 10.1038/mi.2011.7 21451501

[140] Gehring AJ Rojas RE Canaday DH Lakey DL Harding CV Boom WH . The Mycobacterium tuberculosis 19-kilodalton lipoprotein inhibits gamma interferon-regulated HLA-DR and Fc gamma R1 on human macrophages through Toll-like receptor 2. Infect Immun. (2003) 71:4487–97. doi: 10.1128/IAI.71.8.4487-4497.2003 PMC166015 12874328

[141] Park HJ Lee SJ Kim SH Han J Bae J Kim SJ . IL-10 inhibits the starvation induced autophagy in macrophages via class I phosphatidylinositol 3-kinase (PI3K) pathway. Mol Immunol. (2011) 48:720–7. doi: 10.1016/j.molimm.2010.10.020 21095008

[142] O'Leary S O'Sullivan MP Keane J . IL-10 blocks phagosome maturation in mycobacterium tuberculosis-infected human macrophages. Am J Respir Cell Mol Biol. (2011) 45:172–80. doi: 10.1165/rcmb.2010-0319OC 20889800

[143] Duan L Yi M Chen J Li S Chen W . Mycobacterium tuberculosis EIS gene inhibits macrophage autophagy through up-regulation of IL-10 by increasing the acetylation of histone H3. Biochem Biophys Res Commun. (2016) 473:1229–34. doi: 10.1016/j.bbrc.2016.04.045 27079235

[144] Thibodeau J Bourgeois-Daigneault MC Huppe G Tremblay J Aumont A Houde M . Interleukin-10-induced MARCH1 mediates intracellular sequestration of MHC class II in monocytes. Eur J Immunol. (2008) 38:1225–30. doi: 10.1002/eji.200737902 18389477 PMC2759377

[145] Mittal SK Cho KJ Ishido S Roche PA . Interleukin 10 (IL-10)-mediated immunosuppression: MARCH-I induction regulates antigen presentation by macrophages but not dendritic cells. J Biol Chem. (2015) 290:27158–67. doi: 10.1074/jbc.M115.682708 PMC4646393 26408197

[146] Gomez MA Contreras I Halle M Tremblay ML McMaster RW Olivier M . Leishmania GP63 alters host signaling through cleavage-activated protein tyrosine phosphatases. Sci Signal. (2009) 2:ra58. doi: 10.1126/scisignal.2000213 19797268

[147] Abu-Dayyeh I Shio MT Sato S Akira S Cousineau B Olivier M . Leishmania-induced IRAK-1 inactivation is mediated by SHP-1 interacting with an evolutionarily conserved KTIM motif. PloS NeglTrop Dis. (2008) 2:e305. doi: 10.1371/journal.pntd.0000305 19104650 PMC2596967

[148] Everts B Pearce EJ . Metabolic control of dendritic cell activation and function: Recent advances and clinical implications. Front Immunol. (2014) 5:203. doi: 10.3389/fimmu.2014.00203 PMC4021118 24847328

[149] Sukhbaatar N Hengstschlager M Weichhart T . mTOR-mediated regulation of dendritic cell differentiation and function. Trends Immunol. (2016) 37:778–89. doi: 10.1016/j.it.2016.08.009 27614799 PMC6095453

[150] Zeng Q Mallilankaraman K Schwarz H . Increased Akt-driven glycolysis is the basis for the higher potency of CD137L-DCs. Front Immunol. (2019) 10:868. doi: 10.3389/fimmu.2019.00868 31068941 PMC6491642

[151] Jiang Q Qiu Y Kurland IJ Drlica K Subbian S Tyagi S . Glutamine is required for M1-like polarization of macrophages in response to Mycobacterium tuberculosis infection. mBio. (2022) 13:e0127422. doi: 10.1128/mbio.01274-22 35762591 PMC9426538

[152] Cumming BM Addicott KW Adamson JH Steyn AJ . Mycobacterium tuberculosis induces decelerated bioenergetic metabolism in human macrophages. Elife. (2018) 7. doi: 10.7554/elife.39169 30444490 PMC6286123

[153] Saunders EC McConville MJ . Immunometabolism of leishmania granulomas. Immunol Cell Biol. (2020) 98:832–44. doi: 10.1111/imcb.12394 32780446

[154] Pennini ME Liu Y Yang J Croniger CM Boom WH Harding CV . CCAAT/enhancer-binding protein beta and delta binding to CIITA promoters is associated with the inhibition of CIITA expression in response to Mycobacterium tuberculosis 19-kDa lipoprotein. J Immunol. (2007) 179:6910–8. doi: 10.4049/jimmunol.179.10.6910 17982082 PMC2631233

[155] Gay G Braun L Brenier-Pinchart MP Vollaire J Josserand V Bertini RL . Toxoplasma gondii TgIST co-opts host chromatin repressors dampening STAT1-dependent gene regulation and IFN-gamma-mediated host defenses. J Exp Med. (2016) 213:1779–98. doi: 10.1084/jem.20160340 PMC4995087 27503074

[156] Behar SM Divangahi M Remold HG . Evasion of innate immunity by Mycobacterium tuberculosis: is death an exit strategy? Nat Rev Microbiol. (2010) 8:668–74. doi: 10.1038/nrmicro2387 20676146 PMC3221965

[157] Fan L Wu X Jin C Li F Xiong S Dong Y . MptpB promotes mycobacteria survival by inhibiting the expression of inflammatory mediators and cell apoptosis in macrophages. Front Cell Infect Microbiol. (2018) 8:171. doi: 10.3389/fcimb.2018.00171 29888212 PMC5981270

[158] Bogdan C Islam NA Barinberg D Soulat D Schleicher U Rai B . The immunomicrotope of Leishmania control and persistence. Trends Parasitol. (2024) 40:788–804. doi: 10.1016/j.pt.2024.07.013 39174373

[159] Smith-Garvin JE Koretzky GA Jordan MS . T cell activation. Annu Rev Immunol. (2009) 27:591–619. doi: 10.1146/annurev.immunol.021908.132706 19132916 PMC2740335

[160] Weiss A Littman DR . Signal transduction by lymphocyte antigen receptors. Cell. (1994) 76:263–74. doi: 10.1016/0092-8674(94)90334-4 8293463

[161] Macian F . NFAT proteins: key regulators of T-cell development and function. Nat Rev Immunol. (2005) 5:472–84. doi: 10.1038/nri1632 15928679

[162] O'Shea JJ Paul WE . Mechanisms underlying lineage commitment and plasticity of helper CD4+ T cells. Science. (2010) 327:1098–102. doi: 10.1126/science.1178334 PMC2997673 20185720

[163] Stark GR Darnell JE Jr . The JAK-STAT pathway at twenty. Immunity. (2012) 36:503–14. doi: 10.1016/j.immuni.2012.03.013 22520844 PMC3909993

[164] Zhu J Yamane H Paul WE . Differentiation of effector CD4 T cell populations (*). Annu Rev Immunol. (2010) 28:445–89. doi: 10.1146/annurev-immunol-030409-101212 20192806 PMC3502616

[165] Wu G Fan X Cheng L Chen Z Yi Y Liang J . Metabolism-driven posttranslational modifications and immune regulation: emerging targets for immunotherapy. Sci Adv. (2025) 11:eadx6489. doi: 10.1126/sciadv.adx6489 40938975 PMC12428940

[166] Qu J Liu M Zhou C . Rewriting the viral script: post-translational modifications orchestrating SARS-CoV-2 pathogenesis and immune evasion. Front Microbiol. (2026) 17:1748470. doi: 10.3389/fmicb.2026.1748470 41743132 PMC12929506

[167] Chu F Sharma S Ginsberg SD Chiosis G . PTMs as molecular encoders: reprogramming chaperones into epichaperomes for network control in disease. Trends Biochem Sci. (2025) 50:892–905. doi: 10.1016/j.tibs.2025.07.006 40877054 PMC12829026

[168] Indellicato R Trinchera M . Epigenetic regulation of glycosylation in cancer and other diseases. Int J Mol Sci. (2021) 22. doi: 10.3390/ijms22062980 33804149 PMC7999748

[169] Huppa JB Axmann M Mortelmaier MA Lillemeier BF Newell EW Brameshuber M . TCR-peptide-MHC interactions in situ show accelerated kinetics and increased affinity. Nature. (2010) 463:963–7. doi: 10.1038/nature08746 20164930 PMC3273423

[170] Li D Voigt A Nguyen C . Discover potential new epitopes through post-translational modification in Sjogren's disease. Biorxiv. (2025). doi: 10.64898/2025.12.30.697079 41509370 PMC12776321

[171] Shi Y Qin R Li Y . Post-translational modifications: key "regulators" of pancreatic cancer Malignant phenotype-advances in mechanisms and targeted therapies. Biomedicines. (2025) 13. doi: 10.3390/biomedicines13123013 PMC12730280 41463025

[172] Geffen Y Anand S Akiyama Y Yaron TM Song Y Johnson JL . Pan-cancer analysis of post-translational modifications reveals shared patterns of protein regulation. Cell. (2023) 186:3945–67.e3926. doi: 10.1016/j.cell.2023.07.013 37582358 PMC10680287

[173] Jiang W Jaehnig EJ Liao Y Shi Z Yaron-Barir TM Johnson JL . Deciphering the dark cancer phosphoproteome using machine-learned co-regulation of phosphosites. Nat Commun. (2025) 16:2766. doi: 10.1038/s41467-025-57993-2 40113755 PMC11926083

